# The impact of maximal fat oxidation intensity exercise on glucose and lipid metabolism in individuals with overweight or obesity: A systematic review and meta-analysis

**DOI:** 10.5114/biolsport.2026.159565

**Published:** 2026-04-13

**Authors:** Yunyang Liu, Jiawen Gao, Zihan Bao, Ziyang Li, Xunling Wang, Shun Wang

**Affiliations:** 1 Huaibei Normal University, School of Physical Education, Huaibei, China; 2 Shinhan University, Department of Sport and Health, Uijeongbu-si, Republic of Korea

**Keywords:** FATmax training, Overweight and obesity, Meta-analysis, Lipid metabolism, Glucose metabolism, Personalized exercise prescription

## Abstract

This study provides the first quantitative synthesis evaluating the chronic effects of FATmax training on glucose and lipid metabolism in individuals with overweight or obesity, while systematically exploring the moderating roles of participant characteristics and intervention protocols. A comprehensive search of seven databases (e.g., PubMed, Web of Science) was conducted up to August 2025, identifying 24 controlled trials involving 638 participants. Random-effects meta-analyses showed that FATmax training produced significant improvements in multiple glycolipid metabolic markers. Specifically, large standardized reductions were observed in fasting plasma glucose (Hedges’ g = -1.05), insulin resistance (Hedges’ g = -0.82), and fasting insulin (Hedges’ g = -0.75), alongside moderate improvements in triglycerides (Hedges’ g = -0.55), total cholesterol (Hedges’ g = -0.23), and high-density lipoprotein cholesterol (HDL-C; Hedges’ g = 0.51). Notably, the large standardized effects on glycemic markers suggest potentially clinically meaningful improvements in glycemic control. Subgroup analyses indicated that HDL-C adaptations were significantly enhanced in male participants, weight-bearing modalities, and protocols incorporating warm-up sessions or concurrent dietary restriction (p < 0.05). Furthermore, meta-regression identified baseline HDL-C (β= -2.955), exercise intensity (β = 0.053), and session duration (β = 0.058) as significant predictors of HDL-C improvement. Crucially, we derive the first clinically actionable, personalized thresholds from interaction analyses: for individuals with low baseline HDL-C (≤ 1.36 mmol/L), efficacy is maximized when session duration exceeds 60 minutes (Hedges’ g = 1.19) or intensity surpasses 42.2% $\dot{\text{V}}$O_2max_ (Hedges’ g = 1.10); whereas for those with higher baseline levels, extending duration (≥ 60 min) is the primary requisite for significant benefits (Hedges’ g = 0.61). In conclusion, FATmax training produces significant and potentially clinically meaningful improvements in glucose and lipid metabolism in individuals with overweight or obesity. These effects are modulated by individual characteristics and intervention parameters. Future research should prioritize standardized FATmax determination protocols and diverse populations to validate these personalized prescription parameters.

## INTRODUCTION

Overweight and obesity, characterized by excessive accumulation or abnormal distribution of body fat are chronic metabolic disorders arising from the complex interplay of genetic predisposition and environmental influences [1]. In recent decades, these conditions have emerged as severe global public health challenges [2]. Epidemiological data indicate that approximately 2.1 billion adults worldwide are currently overweight or obese, with projections suggesting this figure will rise to 3.3 billion by 2035 [3, 4]. A growing body of evidence demonstrates that excess body fat significantly increases the risk of non-communicable diseases (NCDs) [5], including reduced cardiac and pulmonary function [6], atherosclerosis [7], type 2 diabetes [8], and secondary hypertension [9]. These comorbidities contribute to elevated all-cause mortality and exacerbate the global disease burden.

Exercise is widely recognized as a cornerstone strategy for preventing and managing overweight and obesity [10]. Among various exercise parameters, the scientific selection of aerobic exercise intensity plays a pivotal role in determining the effectiveness of training programs [11]. The intensity corresponding to maximal fat oxidation (FATmax) has gained increasing attention as an individualized prescription for optimizing metabolic regulation. FATmax represents the exercise intensity at which the rate of fat oxidation reaches its peak during incremental workload testing—often referred to as the “golden zone” for maximizing fat utilization [12]. Typically, FATmax falls within the low-to-moderate intensity range, relying primarily on aerobic metabolism. Compared with high-intensity interval training (HIIT) or moderate-intensity continuous training (MICT), FATmax training involves lower intensity and reduced physiological strain while still achieving favorable metabolic and body composition outcomes [13, 14]. Evidence from randomized controlled trials (RCTs) suggests that FATmax training can significantly improve body composition, enhance insulin sensitivity, and optimize lipid and glucose metabolic profiles [15, 16]. Owing to its relatively low physical strain and specific metabolic target, FATmax training is considered a safe and effective intervention for individuals with low fitness levels, limited tolerance to high-intensity exercise, or cardiometabolic impairments.

**Table:** Abbreviations

Hedges’ *g*	Effect size indicator used in pooled analyses
*I* ^2^	Statistical measure of heterogeneity across studies
*K*	Total number of effects included in the pooled analysis
*p*-value	Probability value indicating statistical significance
Power	Statistical power for the pooled effect size
95% CI	95% confidence interval
PI	Prediction interval
GRADE	Grading of Recommendations, Assessment, Development, and Evaluation
CON	Conventional intervention (control)
FATmax	Exercise performed at the individual maximal fat oxidation intensity
RCT	Randomized controlled trial
CT	Controlled trial
TG	Triglycerides
HDL-C	High density lipoprotein cholesterol
LDL-C	Low-density lipoprotein cholesterol
TC	Total cholesterol
FPG	Fasting plasma glucose
HOMA-IR	Homeostasis model assessment of insulin resistance
FINS	Fasting insulin
RT	Resistance training
OW/OB	Participants with overweight or obesity only, without any other comorbid conditions
OW/OB+CD	Participants with overweight or obesity with comorbid chronic disease(s)
NWB-AE	Non-weight-bearing aerobic exercise
WB-AE	Weight-bearing aerobic exercise
BMI	Body mass index
HR at FATmax	Heart rate corresponding to the intensity of maximal fat oxidation
%$\dot{\text{V}}$O_2max_ at FATmax	Percentage of maximal oxygen uptake corresponding to FATmax intensity
$\dot{\text{V}}$O_2_R	Oxygen Uptake Reserve
W	Women
M	Men

Despite the therapeutic potential of FATmax, existing systematic reviews have not fully addressed its impact on metabolic health. To date, only two systematic reviews—by Romain et al. (2012) [17] and Chávez-Guevara et al. (2020) [18]—have systematically evaluated FATmax training. While these reviews provided valuable evidence regarding body weight, fat mass, and cardiorespiratory fitness, they present notable limitations. First, they focused primarily on anthropometric outcomes, failing to quantitatively synthesize the effects of FATmax training on specific glucose and lipid metabolism indicators. Optimizing these metabolic markers is critical for mitigating comorbidities in populations with obesity, yet the specific impact of FATmax on parameters such as triglycerides and insulin resistance remains unclear. Second, previous reviews did not systematically investigate potential moderating factors. Understanding how participant characteristics (e.g., sex, baseline metabolic status) and intervention parameters (e.g., dietary control, session duration) modulate metabolic adaptations is essential for designing precision exercise prescriptions. Furthermore, as new trials have emerged since 2020 [19], an updated synthesis is necessary to refine current knowledge.

To address these critical knowledge gaps, this study aims to provide a comprehensive evaluation of FATmax training with two core novel contributions that distinguish it from prior reviews. First, this is the first quantitative synthesis specifically focused on glycolipid metabolic outcomes (including TG, HDL-C, LDL-C, TC, FPG, HOMAIR, and FINS) rather than solely anthropometric data. Second, this study is the first to provide clinically actionable, personalized thresholds derived from advanced interaction analyses between participant characteristics and intervention protocols. Consequently, our primary objective was to determine the chronic effects of FATmax on metabolic health, while systematically identifying specific dose-response relationships to refine precision exercise prescriptions for individuals with overweight and obesity.

## MATERIALS AND METHODS

This systematic review and meta-analysis was conducted in accordance with the Preferred Reporting Items for Systematic Reviews and Meta-Analyses (PRISMA) 2020 guidelines [20]. A completed PRISMA checklist is provided in Supplementary Table 1. The study protocol was prospectively registered with the PROSPERO database (registration ID: CRD420251111698).

### Information sources

A comprehensive literature search was conducted across PubMed, Web of Science (Core Collection), EBSCOhost, the International Clinical Trials Registry Platform (ICTRP), the Cochrane Library, Embase, and the China National Knowledge Infrastructure (CNKI). The initial search was completed on March 3, 2025, and updated on August 3, 2025. Eligible studies were required to be available in full text, with no restrictions on publication date, population characteristics, or language. For screening feasibility, titles and/or abstracts had to be available in either English or Chinese.

### Search strategy

The search strategy was formulated based on prior systematic reviews and related studies [17, 18, 21, 22]. The following combination of search terms and Boolean operators was applied: (“FATmax” OR “LIPOXmax” OR “FATOXmax” OR “maximal fat oxidation” OR “fat metabolism”) AND (“training” OR “exercise” OR “physical activity”) AND (“obesity” OR “overweight” OR “obese”) AND (“blood glucose” OR “blood lipids” OR “lipid metabolism” OR “glucose metabolism” OR “lipid profile”) AND (“randomized controlled trial” OR “controlled clinical trial” OR “RCT”). Additionally, the PROSPERO registry and Cochrane Database of Systematic Reviews were searched to identify any published or ongoing protocols related to FATmax training. Detailed search strategies and search results for each database are provided in Supplementary Tables 2 and 3. Although multiple terms (e.g., FATmax, LIPOXmax, and FATOXmax) were used in the search strategy to ensure comprehensive retrieval, FATmax was adopted as the primary term throughout the present study, as these terms refer to the same physiological concept of maximal fat oxidation.

### Selection process

All retrieved records were initially deduplicated manually by an independent reviewer (LYY) using EndNote X9 (Clarivate Analytics, Philadelphia, PA, USA). The deduplicated records were then screened independently by two reviewers (GJW and LZY) according to predefined inclusion and exclusion criteria. Screening was conducted in two stages: (1) title and abstract screening and (2) full-text assessment of potentially eligible articles. Discrepancies were resolved through discussion, and unresolved disagreements were adjudicated by a third reviewer (BZH). Interrater reliability was evaluated using Cohen’s *K*, which yielded a coefficient of 0.85, indicating almost perfect agreement [23]. In addition to electronic searches, potentially relevant studies were identified by manually screening the reference lists of prior systematic reviews and by consulting domain experts within the research team.

### Eligibility criteria

Study eligibility was determined according to predefined inclusion and exclusion criteria structured around the PICOS framework (Population, Intervention, Comparison, Outcomes, and Study design). The review included human participants of any age or sex, regardless of their physical activity level. Studies involving animal models were excluded during the title and abstract screening stage.

Eligible studies were required to implement supervised FATmax training interventions without restrictions on training mode, frequency, or duration, provided the protocol lasted at least one week. FATmax was defined as the individualized exercise intensity eliciting the maximal fat oxidation rate. This intensity had to be determined via indirect calorimetry during a graded incremental exercise test with expiratory gas analysis [12, 24]. At each workload stage, fat oxidation rates were calculated, and the intensity yielding the peak oxidation rate was identified as FATmax [25]. To ensure protocol validity, studies utilizing mathematical models to estimate FATmax without direct gas exchange analysis were excluded. Control groups were required to maintain their habitual physical activity levels without engaging in structured exercise programs while adhering to experimental conditions comparable to the intervention group.

Studies were required to report resting and fasting-state data for at least one glycemic or lipid metabolic outcome. Glycemic indicators included fasting plasma glucose (FPG), fasting insulin (FINS), and the homeostatic model assessment of insulin resistance (HOMAIR), while lipid indicators included triglycerides (TG), high-density lipoprotein cholesterol (HDL-C), low-density lipoprotein cholesterol (LDL-C), and total cholesterol (TC). All outcomes had to be measured using standardized laboratory methods. Studies providing sufficient data to calculate effect sizes (e.g., pre- and post-intervention means and standard deviations, or change scores) were considered eligible. Conversely, research focusing exclusively on acute physiological responses (e.g., immediate post-exercise changes) or molecular mechanisms was excluded.

Only original controlled trials employing between-group comparisons (parallel or crossover), whether randomized or non-randomized, were included. Acute exercise trials, review articles, editorials, commentaries, validation studies, book chapters, and case reports were excluded.

### Data extraction

Two reviewers (GJW and LZY) independently extracted data using a pre-specified worksheet developed in Microsoft Excel (Microsoft 365; Microsoft, Redmond, WA, USA). Extracted information included the first author’s name, publication year, study design, participant characteristics, training protocol details, dropout rate, and pre- and post-intervention means and standard deviations for all relevant outcome variables. Group sample sizes were also recorded. When essential data were incomplete or presented graphically, the corresponding authors were contacted for clarification. If no response was obtained, numerical data were extracted from figures using WebPlotDigitizer (version 5.2; https://automeris.io/) [26]. Studies were excluded if critical outcome data could not be retrieved or accurately estimated.

### Risk of bias assessment

The risk of bias for randomized controlled trials was evaluated using the Cochrane Risk of Bias 2.0 (RoB 2.0) tool [27], which assesses five domains: (1) bias arising from the randomization process, (2) deviations from intended interventions, (3) missing outcome data, (4) outcome measurement, and (5) selection of the reported result. Disagreements between reviewers were resolved through discussion, and any unresolved issues were adjudicated by a third reviewer (BZH). For non-randomized studies, the Risk of Bias in Non-randomized Studies of Interventions (ROBINS-I) tool [27] was employed to assess seven domains: confounding, participant selection, intervention classification, deviations from intended interventions, missing data, outcome measurement, and selection of the reported result. Additionally, methodological quality was evaluated using the Physiotherapy Evidence Database (PEDro) scale (0–10 points) [28]. Scores of ≥ 6 were considered high quality, 4–5 moderate quality, and ≤ 3 low quality.

### Statistical analysis

#### Data synthesis and effect measures

Between-group comparisons of the FATmax and control interventions were conducted using change scores (mean difference and standard deviation) derived from pre- and post-intervention data, calculated according to established equations [29–31]. The mean difference (*M*_diff_) was first computed as:


$${\text{M}}_{\text{diff}}={\text{M}}_{\text{post}}-{\text{M}}_{\text{pre}}$$

where *M*_diff_ represents the raw mean difference, *M*_post_ is the postintervention mean, and *M*_pre_ is the pre-intervention mean [32].

The standard deviation of the change in means SD_diff_ was then estimated using the formula:


$${\text{SD}}_{\text{diff}}=\sqrt{{\text{SD}}_{\text{pre}}^{2}+{\text{SD}}_{\text{post}}^{2}-2\text{r}\times {\text{SD}}_{\text{pre}}\times {\text{SD}}_{\text{post}}}$$

where SD_diff_ denotes the standard deviation of the change scores, SD_pre_ and SD_post_ refer to the pre- and post-intervention standard deviations, and *r* represents the pre-post correlation coefficient [32]. Since most included trials did not report this coefficient, a conservative value of *r* = 0.50 was adopted. This approach aligns with the Cochrane Handbook guidelines [32], established statistical recommendations [33], and comparable meta-analyses in the field of exercise physiology [17, 18, 34]. To ensure the stability of our findings, the robustness of the pooled estimates was further validated through sensitivity analyses using alternative coefficients (*r* = 0.25 and 0.75), as detailed in Section 3.6.

Given that several included studies involved relatively small sample sizes, Hedges’ *g* was used as the effect size estimator, calculated as follows [35]:


$$\begin{array}{l} \text{Hedge’g}=\frac{\left(\text{FATmax}({\text{M}}_{\text{diff}})-\text{CON}({\text{M}}_{\text{diff}})\right)}{{\text{SD}}_{\text{pooled}}}\\ \;\times (1-\frac{3}{4({\text{n}}_{1}+{\text{n}}_{2}-2)-1}) \end{array}$$

Here, FATmax (*M*_diff_) and CON (*M*_diff_) denote the mean change in the FATmax and control groups, respectively; *n*_1_ and *n*_2_ indicate the sample sizes of the two groups; and SD_pooled_ is the pooled standard deviation, computed as:


$${\text{SD}}_{\text{pooled}}=\sqrt{\frac{({\text{n}}_{1}-1)\times {\text{SD}}_{1}^{2}+({\text{n}}_{2}-1)+{\text{SD}}_{2}^{2}}{\left({\text{n}}_{1}+{\text{n}}_{2}-2\right)}}$$

where SD_1_ and SD_2_ are the standard deviations of the FATmax and control groups, respectively. According to conventional interpretation thresholds, Hedges’ *g* values were classified as follows: trivial (< 0.2), small (0.2–0.5), medium (> 0.5–0.8), and large (> 0.8) [36].

#### Subgroup and meta-regression analysis

Between-study heterogeneity was quantified using the *I*^2^ statistic and interpreted according to the Cochrane Handbook thresholds, where 0–40% may not be important, 30–60% represents moderate heterogeneity, 50–90% indicates substantial heterogeneity, and 75–100% suggests considerable heterogeneity [32]. In line with general methodological recommendations, when substantial heterogeneity was detected (*I*^2^ > 50%), subgroup and meta-regression analyses were conducted under a random-effects framework to explore potential sources of variation [37, 38]. To reduce the likelihood of false-negative findings due to limited power, the statistical power for each subgroup was also calculated [39].

Based on prior evidence, the following categorical variables were examined in the subgroup analyses: (a) participant sex, (b) inclusion of a warm-up session, (c) inclusion of dietary control, (d) presence of co-morbid chronic diseases, (e) training modality, and (f) randomization status.

To further elucidate the dose – response relationship between FATmax training and glycolipid metabolism, mixed-effects meta-regression analyses were conducted using restricted maximum likelihood (REML) estimation [40]. Moderators were selected a priori based on physiological plausibility, specifically focusing on exerciseinduced metabolic adaptations (e.g., time-dependent lipid oxidation kinetics) and the law of initial values. Accordingly, the models incorporated participant characteristics (age, baseline BMI, baseline HDLC, baseline FPG, and baseline $\dot{\text{V}}$O_2max_) and key training parameters, including session duration (minutes), training frequency (days/week), intervention duration (weeks), total training volume (MET-min/week), and exercise adherence (%).

Both linear and cubic functions were fitted and compared to characterize the dose – response pattern. The model with the lowest bias-corrected Akaike Information Criterion (AIC) value was selected as the best-fitting model [41]. All analyses were conducted using the metafor package (version 3.0; Wolfgang Viechtbauer, Maastricht University), and visualizations were produced using ggplot2 (version 3.3.6; Hadley Wickham, RStudio, USA) [42].

#### Risk of publication bias and sensitivity analysis

Publication bias was assessed visually using contour-enhanced funnel plots [43] and quantitatively via Egger’s linear regression test [44, 45]. In accordance with established guidelines, potential asymmetry was interpreted with caution, acknowledging that it may arise from between-study heterogeneity or chance (small-study effects) rather than solely from publication bias [46]. A *p*-value > 0.05 in Egger’s test, combined with a symmetrical funnel plot, was interpreted as indicating no significant risk of publication bias.

To verify the robustness of our findings, we employed a three-tiered sensitivity analysis. First, we evaluated the impact of methodological assumptions regarding data imputation. We recalculated the pooled effects using alternative correlation coefficients (*r* = 0.25 and 0.75) to determine if the results were sensitive to the assumed value (*r* = 0.50). Additionally, we compared the results derived from the SD of change scores against those using baseline SDs. Second, a leave-oneout analysis was conducted by sequentially excluding individual studies to detect whether any single trial disproportionately skewed the aggregate effect size. Third, we screened for influential outliers within the multilevel meta-analytic framework. Cook’s distance [47] and studentized residuals [48] were utilized to diagnose leverage and influential cases at both the within-study (Level 2) and between-study (Level 3) levels. Data points were flagged as outliers if their hat values or Cook’s distances exceeded three times the mean, or if absolute studentized residuals were greater than 3. The meta-analysis models were subsequently re-estimated after excluding these flagged cases to confirm the stability of the primary outcomes.

### Certainty of the evidence

The GRADE (Grading of Recommendations Assessment, Development and Evaluation) approach was applied to integrate the risk of bias into the interpretation of results and to rate the certainty of evidence as high, moderate, low, or very low. In brief, high certainty indicates that further research is very unlikely to change confidence in the estimated effect; moderate certainty suggests that additional research may have an important impact on confidence in the estimate and could alter it; low certainty denotes that further research is likely to have an important impact and may change the estimate; and very low certainty implies that the true effect is highly uncertain [49]. All GRADE assessments were independently performed by one reviewer (GJW) and verified by a second reviewer (WS).

## RESULTS

### Studies retrieved

**FIG. 1:**
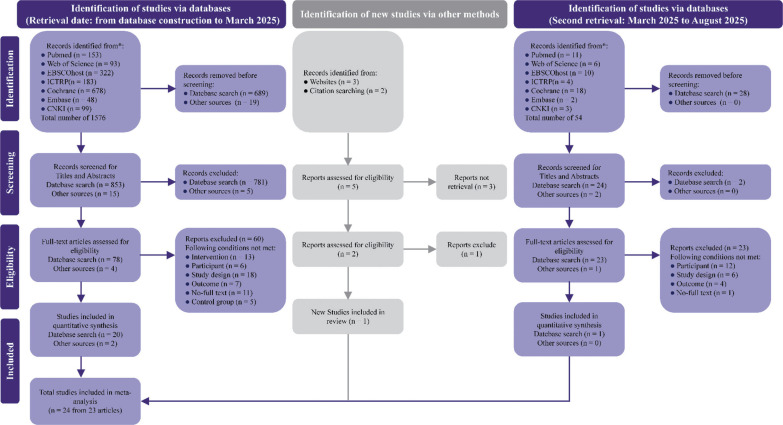
PRISMA flow diagram for included and excluded studies. Note: n represents the number of studies.

The search identified 1,635 records: 1,576 from the primary database search, 54 from an update conducted six months later, and 1 from other sources. After screening, 24 studies were included in the meta-analysis [15, 16, 19, 50–69] (Fig. 1).

### Characteristics of included studies

Of the 24 included studies, 16 were randomized controlled trials (R CTs) [15, 19, 50–57, 59, 62–64, 67, 69], and 8 were non-randomized controlled trials [16, 58, 60, 61, 65, 66, 68]. The pooled sample comprised 638 participants (193 males, 323 females, and 122 with unreported sex). Sample sizes across studies ranged from 8 to 68 participants, with participant ages ranging from 13 to 64 years. Detailed characteristics of participants and intervention protocols are summarized in Supplementary Table 4.

### Primary analysis

**FIG. 2:**
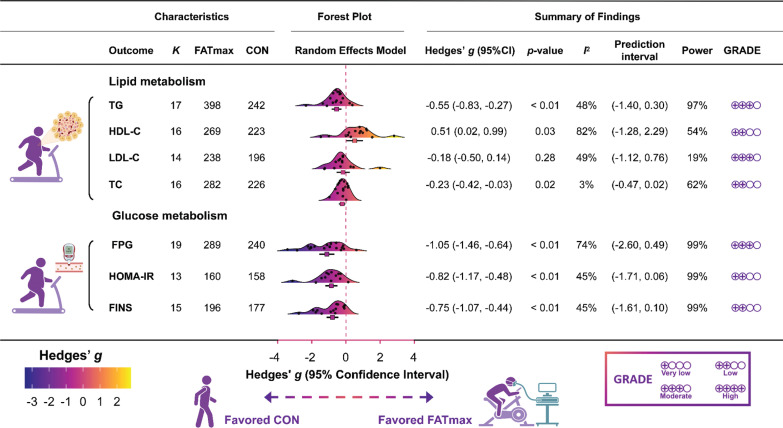
Pooled effect sizes for the primary outcomes.

Compared with conventional interventions, FATmax training was associated with significant improvements in lipid metabolism among individuals with overweight or obesity. Specifically, FATmax training significantly reduced triglyceride (TG) levels (*k* = 17, *g* = -0.55, 95% CI: -0.83 to -0.27, *I*^2^ = 48% [moderate], PI: -1.40 to 0.30, *p* < 0.01; moderate certainty) and total cholesterol (TC) (*k* = 16, *g* = -0.23, 95% CI: -0.42 to -0.03, *I*^2^ = 3% [low], PI: -0.47 to 0.02, *p* = 0.02; low certainty), while significantly increasing highdensity lipoprotein cholesterol (HDL-C) (*k* = 16, *g* = 0.51, 95% CI: 0.02 to 0.99, *I*^2^ = 82% [substantial], PI: -1.28 to 2.29, *p* = 0.03; low certainty). In contrast, no significant change was observed in low-density lipoprotein cholesterol (LDL-C) (*k* = 14, *g* = -0.18, 95% CI: -0.50 to 0.14, *I*^2^ = 49% [moderate], PI: -1.12 to 0.76, *p* = 0.28; moderate certainty) (Fig. 2).

FATmax training also elicited significant improvements in glucose homeostasis relative to conventional interventions. Notably, fasting plasma glucose (FPG) decreased significantly (*k* = 19, *g* = -1.05, 95% CI: -1.46 to -0.64, *I*^2^ = 74% [substantial], PI: -2.60 to 0.49, *p* < 0.01; moderate certainty), as did the homeostatic model assessment for insulin resistance (HOMA-IR) (*k* = 13, *g* = -0.82, 95% CI: -1.17 to -0.48, *I*^2^ = 45% [moderate], PI: -1.71 to 0.06, *p* < 0.01; low certainty) and fasting insulin (FINS) (*k* = 15, *g* = -0.75, 95% CI: -1.07 to -0.44, *I*^2^ = 45% [moderate], PI: -1.61 to 0.10, *p* < 0.01; low certainty) (Fig. 2).

Detailed forest plots for each outcome are presented in Supplementary Fig. 2–5, and the statistical power of the pooled results is summarized in Supplementary Fig. 6.

### Moderator analysis

**FIG. 3:**
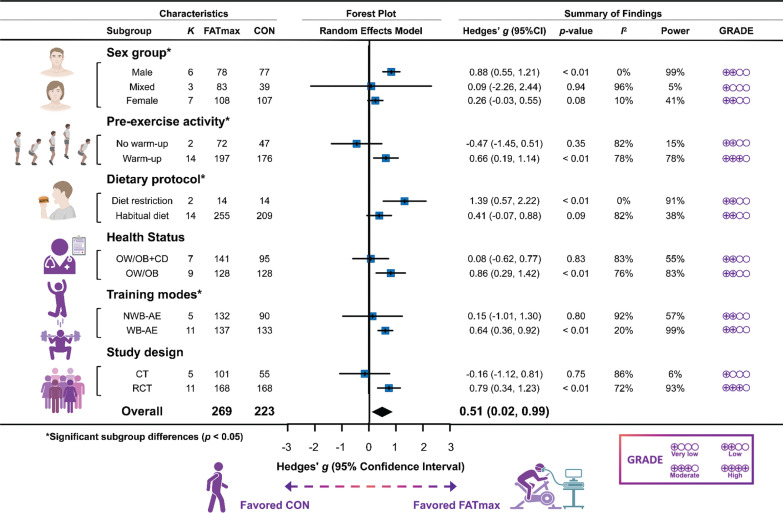
Subgroup analyses based on HDL-C.

Owing to the substantial heterogeneity observed in HDL-C and FPG outcomes, moderator analyses were performed to explore the influence of participant characteristics, pre-exercise activity, dietary control, and training modality on HDL-C (Fig. 3) and FPG (Supplementary Fig. 7).

#### Potential moderators of HDL-C

Subgroup analyses revealed that sex, pre-exercise activity status, dietary protocol, and training modality significantly moderated the effects of FATmax training on HDL-C levels in individuals with overweight or obesity (*p* < 0.05, Fig. 3). Specifically, FATmax training elicited greater increases in HDL-C among men (*g* = 0.88), in studies incorporating a warm-up session (*g* = 0.66), when combined with dietary restriction (*g* = 1.39), and in weight-bearing aerobic exercise (WB-AE, e.g., running) (*g* = 0.64) compared to non-weight-bearing aerobic exercise (NWB-AE, e.g., cycling) (*g* = 0.15). However, given the limited number of studies (*k*) and participants (n) included in the “Male” and “Dietary restriction” subgroups, these specific findings should be regarded as exploratory. The substantial effect sizes observed in these subsets must be interpreted with caution and warrant validation through larger-scale trials to ensure statistical robustness. Regarding other potential moderators, no statistically significant subgroup differences were observed with respect to participants’ health status or study design. Nevertheless, FATmax training produced significantly greater improvements in HDL-C compared with controls under specific conditions. Notably, within the subgroup of simple overweight or obesity (OW/OB), FATmax training conferred a clear advantage (*g* = 0.86), whereas the effect in individuals with overweight or obesity combined with chronic diseases (OW/OB+CD) was not statistically significant (*g* = 0.08). Similar benefits were observed in randomized controlled trials (RCT) (*g* = 0.79).

**FIG. 4:**
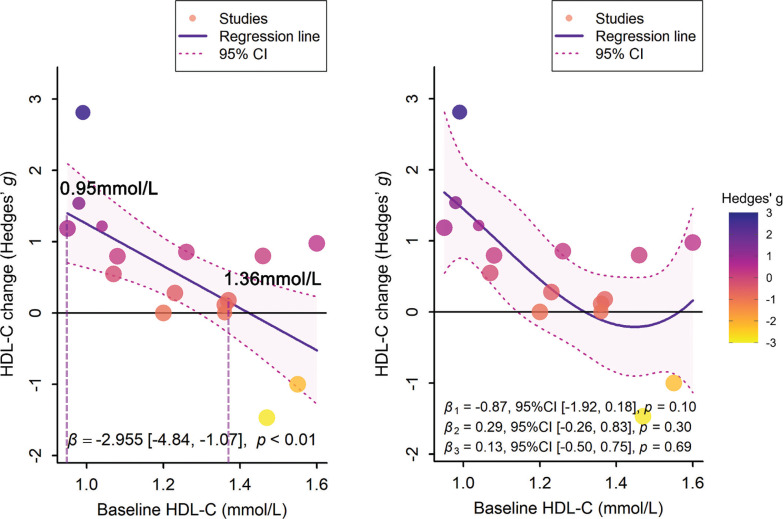
Meta-regression analysis of HDL-C changes in relation to baseline HDL-C levels. The horizontal axis represents baseline HDL-C (0.95–1.36 mmol/L), and the vertical axis represents the change in HDL-C before and after the intervention, with each bubble corresponding to one study. The left panel displays the simple linear regression model, whereas the right panel illustrates the cubic regression model. The value of 1.36 mmol/L indicates the upper significant threshold for the model prediction. When baseline HDL-C ≤ 1.36 mmol/L, post-intervention increases in HDL-C are more likely to reach statistical significance. Comparison of Akaike information criterion (AIC) values indicated that the linear regression model (left panel) provided a relatively better fit.

**FIG. 5:**
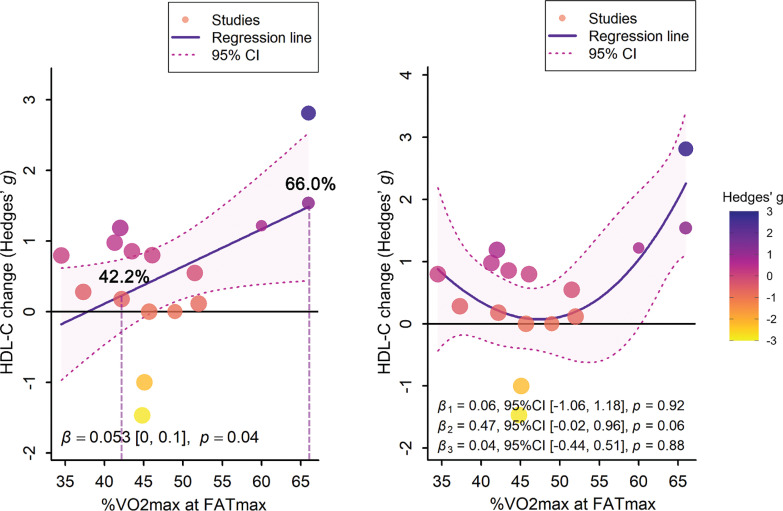
Meta-regression analysis of HDL-C changes in relation to %$\dot{\text{V}}$O_2max_ at FATmax. The horizontal axis represents %$\dot{\text{V}}$O_2max_ at FATmax (42.2%–66.0%), and the vertical axis represents the change in HDL-C before and after the intervention, with each bubble corresponding to one study. The left panel displays the simple linear regression model, whereas the right panel illustrates the cubic regression model. The value of 42.2% indicates the lower significant threshold for the model prediction. When %$\dot{\text{V}}$O_2max_ at FATmax ≥ 42.2%, post-intervention increases in HDL-C are more likely to reach statistical significance. Comparison of Akaike information criterion (AIC) values indicated that the linear regression model (left panel) provided a relatively better fit.

**FIG. 6:**
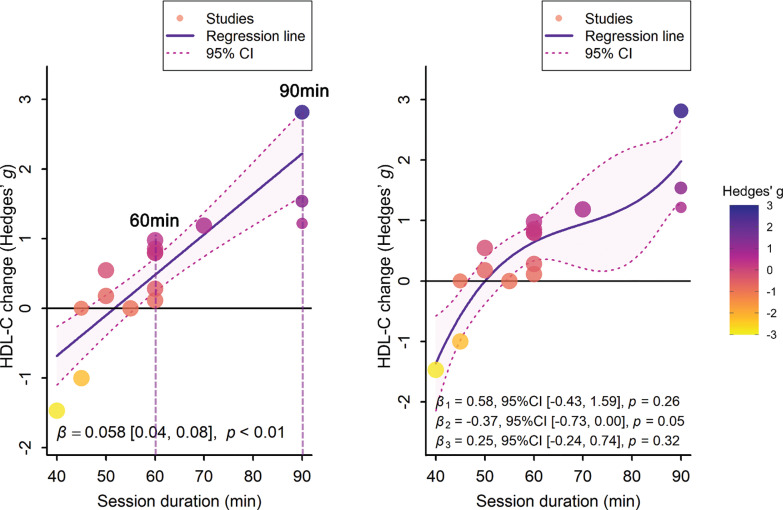
Meta-regression analysis of HDL-C changes in relation to session duration. The horizontal axis represents session duration (60–90 min), and the vertical axis represents the change in HDL-C before and after the intervention, with each bubble corresponding to one study. The left panel displays the simple linear regression model, whereas the right panel illustrates the cubic regression model. The value of 60 min indicates the lower significant threshold for the model prediction. When session duration ≥ 60 min, postintervention increases in HDL-C are more likely to reach statistical significance. Comparison of Akaike information criterion (AIC) values indicated that the linear regression model (left panel) provided a relatively better fit.

Meta-regression analyses identified baseline HDL-C concentration, %$\dot{\text{V}}$O_2max_ at FATmax, and session duration as significant predictors of HDL-C improvement. The linear model demonstrated superior fit according to the Akaike Information Criterion. Specifically: (1) Baseline HDL-C: When baseline HDL-C ≤ 1.36 mmol/L, lower initial HDL-C levels were associated with greater post-intervention increases (β = -2.955, 95% CI: -4.84 to -1.07; *p* < 0.01). Within the range of 0.95–1.36 mmol/L, each 1 mmol/L increase in baseline HDL-C was associated with an average reduction of approximately 0.71 mmol/L in the intervention benefit (β = -0.71, 95% CI: -1.16 to -0.26; *p* < 0.01), indicating a ceiling effect in individuals with higher baseline HDL-C (Fig. 4). (2) Exercise intensity: When %$\dot{\text{V}}$O_2max_ at FATmax ≥ 42.2%, higher training intensity was associated with greater HDL-C improvements (β = 0.053, 95% CI: 0.00 to 0.10; *p* = 0.04). Within the 42.2–66% $\dot{\text{V}}$O_2max_ range, each 1% increase in exercise intensity yielded an additional HDL-C gain of approximately 0.013 mmol/L (β = 0.013, 95% CI: 0.00 to 0.03; *p* = 0.04) (Fig. 5). (3) Session duration: When training sessions lasted ≥ 60 minutes, longer durations were linked to greater HDL-C enhancements (β = 0.058, 95% CI: 0.04 to 0.08; *p* < 0.01). Within the 60–90-minute range, each additional minute of exercise was associated with an HDL-C increase of approximately 0.014 mmol/L (β = 0.014, 95% CI: 0.01 to 0.02; *p* < 0.01) (Fig. 6). No significant moderating effects were found for age, BMI, baseline cardiorespiratory fitness ($\dot{\text{V}}$O_2max_>), baseline FPG, heart rate, training frequency, intervention duration, total training volume (MET-min/week), or exercise adherence (*p* > 0.05, Supplementary Fig. 8).

To enhance clinical applicability, a combined subgroup analysis explored the interaction effects of these significant moderators (Supplementary Fig. 9). Regarding the interaction between baseline HDLC and session duration (Pattern 1), results for the low HDL-C subgroup (≤ 1.36 mmol/L) showed that employing a long session duration (≥ 60 min) yielded robust and clinically meaningful improvements (*g* = 1.19), whereas a short session duration (< 60 min) proved ineffective (*p* = 0.43). Similarly, the high HDL-C subgroup (> 1.36 mmol/L) also required a long session duration to achieve significant benefits (*g* = 0.61). In terms of the interaction between baseline HDL-C and exercise intensity (Pattern 2), the low HDL-C subgroup responded positively to both intensities but derived greater benefit from high intensity (≥ 42.2% $\dot{\text{V}}$O_2max_; *g* = 1.10) compared to low intensity (< 42.2% $\dot{\text{V}}$O_2max_; *g* = 0.56). In contrast, high intensity failed to elicit significant changes in the high HDL-C subgroup. Finally, the interaction between duration and intensity (Pattern 3) revealed that session length was the primary driver of efficacy. Long session durations consistently produced significant improvements regardless of whether low intensity (*g* = 0.76) or high intensity (*g* = 1.18) was prescribed. Conversely, short session durations did not yield significant benefits, even when combined with high exercise intensity (*p* = 0.29). Collectively, these findings suggest that prioritizing extended session durations is critical for optimizing HDLC, particularly for individuals with low baseline levels or when implementing high-intensity FATmax training.

#### Potential moderators of FPG

Across all subgroups, no statistically significant differences were observed for fasting plasma glucose (FPG) (*p* > 0.05 for all comparisons; Supplementary Fig. 7). However, FATmax training demonstrated a significant advantage over the control group in reducing FPG levels under most conditions. In the sex subgroups, males (*g* = -0.97), females (*g* = -1.01), and mixed samples (*g* = -1.69) all exhibited significant reductions, suggesting that sex was not a determining factor in the improvement of FPG. Regarding pre-exercise activity, only studies incorporating a warm-up session showed significant decreases in FPG (*g* = -1.16), whereas those without a warm-up did not reach statistical significance (*p* = 0.07). For dietary protocols, both dietary restriction (*g* = -1.74) and habitual diet conditions (*g* = -1.01) were associated with significant reductions. In terms of health status, significant improvements were observed in the simple overweight or obesity (OW/OB) subgroup (*g* = -1.00) and the overweight or obesity combined with chronic diseases (OW/OB+CD) subgroup (*g* = -1.25), indicating consistent efficacy across populations. With respect to training mode, both non-weight-bearing aerobic exercise (NWB-AE) (*g* = -1.38) and weight-bearing aerobic exercise (WB-AE) (*g* = -0.87) produced significant improvements in FPG. Furthermore, both randomized controlled trials (RCT; *g* = -0.96) and controlled trials (CT; *g* = -1.34) demonstrated significant reductions. Collectively, although no statistically significant subgroup differences were detected, FATmax training effectively reduced FPG across a wide range of conditions and participant characteristics, with effect sizes generally indicating large clinical magnitudes (*g* > 0.8).

Furthermore, meta-regression analysis revealed no significant moderating effects of age, BMI, baseline cardiorespiratory fitness ($\dot{\text{V}}$O_2max_), baseline FPG, %$\dot{\text{V}}$O_2max_ at FATmax, heart rate at FATmax, session duration, training frequency, intervention duration, total training volume (MET-min/week), or exercise adherence on the relationship between FATmax training and FPG (*p* > 0.05 for all comparisons; Supplementary Fig. 10).

### Risk of bias and quality of methods

Most included studies were rated as having “some concerns,” as illustrated in Supplementary Fig. 11. According to the RoB 2.0 assessment, the most frequent sources of bias were associated with the randomization process (Domain 1), deviations from intended interventions (Domain 2), and selective reporting (Domain 5). In contrast, low risks of bias were observed for missing outcome data (Domain 3) and outcome measurement (Domain 4), reflecting consistent data completeness and standardized measurement procedures across studies. In the ROBINS-I assessment, non-randomized studies exhibited higher risks of bias, primarily related to confounding factors and participant selection. Notably, studies by Mohebbi et al. [61], Dumortier et al. [65], and Maurie et al. [66] showed elevated risk levels in these domains. It is crucial to acknowledge that these methodological limitations may have influenced the overall pooled estimates. Specifically, the lack of randomization introduces the potential for self-selection bias, where participants in the intervention groups might possess higher intrinsic motivation or better baseline health behaviors than those in the control groups. Such baseline imbalances could theoretically inflate the observed metabolic improvements, potentially leading to a slight overestimation of the pooled effect sizes for FATmax efficacy compared to strictly randomized conditions. While these issues appeared to arise largely from insufficient methodological reporting rather than substantive flaws in implementation, the contribution of these non-randomized trials to the aggregate results should be interpreted with this potential upward bias in mind.

Potential publication bias was evaluated using funnel plot asymmetry in conjunction with Egger’s regression test for outcomes including TG, HDL-C, LDL-C, TC, FPG, HOMA-IR, and FINS (Supplementary Fig. 12). Egger’s test indicated possible publication bias for TG (*p* < 0.01), HDL-C (*p* = 0.01), and FPG (*p* < 0.01), whereas no significant bias was detected for TC (*p* = 0.47), LDL-C (*p* = 0.10), HOMA-IR (*p* = 0.15), and FINS (*p* = 0.10).

Overall methodological quality was rated as moderate to high, with a mean PEDro score of 5.9 across the included studies (Supplementary Table 5), suggesting that despite some reporting limitations, the evidence base remains methodologically sound and suitable for quantitative synthesis.

### Sensitivity analysis

Sensitivity analyses confirmed the robustness of the primary findings across different methodological assumptions. First, analyses employing baseline standard deviations (SDs) yielded statistical significance (*p*-values) consistent with the primary analysis across all outcomes, with only marginal variations observed in heterogeneity estimates (Supplementary Table 6). Second, to verify the stability of the effect size estimation, sensitivity analyses were conducted using alternative pre-post correlation coefficients (*r* = 0.25 and 0.75). The results demonstrated that the pooled effect sizes remained robust to these variations, indicating that the adoption of a conservative coefficient (*r* = 0.50) did not introduce bias to the overall conclusions.

Furthermore, leave-one-out sensitivity analyses were conducted for all primary outcomes (Supplementary Figs. 13–14). The results indicated that for outcomes with lower statistical power, the exclusion of individual studies exerted a more pronounced influence on the pooled estimates. Specifically, in the analysis of LDL-C, the exclusion of Dumortier et al. [65] (*k* = 13, *g* = -0.25, *p* < 0.05) shifted the overall effect from non-significant to significant.

Potential outliers were identified within the multilevel meta-analytic framework using Cook’s distance and studentized residuals. Outliers were detected for HDL-C, fasting plasma glucose (FPG), HOMAIR, and fasting insulin (FINS). Specifically, Dumortier et al. [65] and Maurie et al. [66] were flagged as outliers for HDL-C based on Cook’s distance; however, their exclusion did not meaningfully alter the repooled estimate (*k* = 14, *g* = 0.73, *p* < 0.01). Similarly, Safarimosavi et al. [63] was identified as an influential outlier for FPG, HOMAIR, and FINS by both diagnostic metrics, yet its exclusion did not materially affect the pooled results (FPG: *k* = 18, *g* = -0.96, *p* < 0.01; HOMA-IR: *k* = 12, *g* = -0.69, *p* = 0.11; FINS: *k* = 14, *g* = -0.62, *p* < 0.01).

Collectively, these specific studies [63, 65, 66] appear to be significant contributors to the heterogeneity observed in HDL-C and FPG. This divergence may be attributed to two primary factors. First, although Dumortier et al. [65] and Maurie et al. [66] both utilized lipid oxidation-based training prescriptions, methodological discrepancies in FATmax determination, target intensity setting, and individualized threshold definitions likely resulted in inconsistent intervention dosages and metabolic responses, thereby amplifying variability in HDL-C and FPG outcomes. Second, Safarimosavi et al. [63] recruited male participants with prediabetes who exhibited lower baseline glucose levels and potentially higher metabolic plasticity, leading to substantial post-intervention improvements in glycemic and insulin indices. These characteristics likely contributed to the inflated effect sizes for FPG and HOMA-IR observed in that study. Crucially, however, the exclusion of these outliers did not fundamentally change the overall conclusions for HDL-C and FPG, thereby confirming the robustness and stability of the findings.

## DISCUSSION

This study demonstrates that FATmax training confers significant metabolic benefits in individuals with overweight or obesity. While previous meta-analyses by Romain et al. [17] and Chávez-Guevara et al. [18] established the efficacy of FATmax for anthropometric adaptations and cardiorespiratory fitness, the present study advances the field by shifting focus toward clinical utility and analytical depth. Unlike prior reviews limited to body composition, this investigation provides the first comprehensive quantitative synthesis specifically targeting glycolipid metabolic markers (e.g., FPG, HOMA-IR, and lipid fractions). Furthermore, we extend beyond aggregate effects to systematically explore dose-response interactions, identifying precise modulators of efficacy. These findings fill a critical gap in precision exercise prescription by establishing personalized intensity and duration thresholds. Notably, the intervention elicited large reductions in key glucose homeostasis markers (FPG, HOMA-IR, and FINS), offering clinical value beyond mere statistical significance. While FATmax also optimized lipid profiles (decreased TG/TC, increased HDL-C), the magnitude of these adaptations is nuanced, largely modulated by participant characteristics and intervention protocols— particularly intensity, modality, and duration.

### Metabolic Effects of FATmax Training

#### Lipid metabolism capacity

Precise prescription of exercise intensity is a critical determinant of improvements in lipid metabolism among individuals with overweight or obesity. Unlike conventional aerobic training that primarily emphasizes total energy expenditure, training at the fat oxidation maximal zone (FATmax) specifically targets the metabolic range corresponding to an individual’s maximal fat oxidation rate (MFO), thereby enhancing the efficiency of lipid mobilization and oxidation [12]. Consequently, FATmax training offers unique metabolic advantages, particularly in regulating lipid fractions tied to oxidative efficiency.

The results of this meta-analysis demonstrate that FATmax training significantly improves key lipid parameters, specifically driving robust reductions in total cholesterol (TC) and triglycerides (TG), alongside significant increases in high-density lipoprotein cholesterol (HDL-C). However, the lack of significant reduction in LDL-C highlights a fundamental mechanistic divergence between the pathways optimized by FATmax and those required for effective LDL clearance. On one hand, FATmax primarily modulates peripheral lipid processing; by targeting maximal fat oxidation, it enhances lipoprotein lipase (LPL) activity, which accelerates the hydrolysis of TG-rich lipoproteins and fuels HDL-mediated reverse cholesterol transport [70, 71]. In stark contrast, the clearance of circulating LDL-C relies predominantly on hepatic mechanisms, specifically the upregulation of hepatic LDL receptors (LDLR) [72]. While pharmacological interventions (e.g., statins) and high-intensity aerobic exercise effectively stimulate LDLR expression to lower LDL-C [73, 74], FATmax appears to lack the requisite physiological stress or systemic threshold to activate this hepatic pathway [70, 75]. Consequently, while FATmax is highly effective for metabolic regulation at the muscular and peripheral level [24], it does not mimic the LDLR-mediated clearance mechanisms typically induced by pharmacotherapy or higherintensity training, leaving LDL-C levels largely unaffected [70, 75].

Sex-specific physiological characteristics appear to modulate lipid adaptations following FATmax training. Our analysis indicates that men generally achieved greater improvements in HDL-C. This trend may be attributed to higher lipoprotein lipase expression and elevated fatty acid uptake rates in males [76, 77]. Nevertheless, we emphasize that the limited number of male-only cohorts constrains the statistical power of this subgroup analysis. Consequently, these observed sex differences must be viewed as exploratory rather than definitive outcomes and warrant validation through larger and more balanced investigations. Similarly, interpretations regarding the combined effects of FATmax exercise and dietary energy restriction require caution. While the data suggest that combining these interventions enhances weight loss and lipid profiles beyond exercise alone [13, 78], the statistical power for this specific subgroup remains limited due to the small number of included studies. Therefore, these findings should be considered preliminary. However, regarding health status, our subgroup analysis revealed that significant HDL-C improvements were concentrated in individuals with simple overweight or obesity, whereas those with comorbid chronic diseases (e.g., type 2 diabetes) did not exhibit statistically significant changes. This disparity suggests that established metabolic pathologies may induce a state of metabolic inflexibility [79], where the physiological mechanisms for lipid oxidation and reverse cholesterol transport are impaired [80]. Consequently, this dysfunction may dampen the lipid-modulating efficacy of FATmax training in these specific populations [81]. Pre-exercise preparation also influences FATmax efficacy. Gray et al. demonstrated that an active warm-up enhances lipid oxidation and metabolic readiness [82]. Thus, gradually increasing exercise intensity to reach the FATmax range may more effectively activate lipid mobilization compared with starting directly at the target intensity. Finally, concerning exercise modality, our subgroup analyses indicated that weightbearing aerobic exercises (WB-AE), such as running, elicited significantly greater HDL-C improvements compared to non-weight-bearing aerobic exercises (NWB-AE), such as cycling. While Achten et al. observed no significant differences in acute fat oxidation rates between modalities under matched intensities [83], Thomas et al. found that treadmill exercise induced approximately 21% greater fat oxidation than cycling [84]. This advantage of WB-AE likely reflects the greater muscle mass recruitment and mechanical load required to support body weight, which drives higher total energy expenditure and systemic lipid mobilization. Therefore, when orthopedic conditions permit, clinicians might prioritize weight-bearing modalities to maximize metabolic adaptations.

Unlike traditional aerobic protocols that primarily rely on total volume, FATmax-induced adaptations hinge on achieving the specific metabolic threshold for maximal fat oxidation [12, 13]. Our regression analysis identified a critical intensity threshold of approximately 42.2% $\dot{\text{V}}$O_2max_. Although this value aligns with the lower boundary of moderate intensity (40–59% $\dot{\text{V}}$O_2_R) recommended by the American College of Sports Medicine (ACSM) for weight management [85, 86], our findings provide a necessary stratification to enhance clinical precision. Specifically for individuals presenting with low baseline HDL levels (≤ 1.36 mmol/L), adherence to this intensity threshold appears decisive, eliciting therapeutic benefits of clinical significance that exceed those observed at lower intensities. This indicates that for this specific phenotype, the vigorous intensity zones often prescribed for fitness conditioning may be superfluous. Instead, maintaining intensity within the FATmax optimal zone (typically 40–50% $\dot{\text{V}}$O_2max_) optimizes the balance between metabolic efficiency and physiological tolerability, thereby maximizing efficacy for the population most in need of lipid remodeling.

From a clinical perspective, session duration constitutes a pivotal determinant for optimizing lipid profiles, suggesting that generalized protocols may require specific refinement for effective obesity management. Although the American College of Sports Medicine (ACSM) recommends accumulating a minimum of 150 minutes of moderate-intensity physical activity per week for general health [86], our analysis indicates that this standard volume is often inadequate for driving robust HDL cholesterol remodeling in individuals with obesity. We observed that while therapeutic efficacy was influenced by baseline lipid status, the necessity for prolonged duration appeared universal. Specifically, for individuals with low baseline HDL levels, extending sessions beyond 60 minutes was obligatory to elicit substantial improvements, whereas shorter durations failed to trigger comparable adaptations. Furthermore, even for those with higher baseline levels, prolonged duration served as the primary requisite for maximizing benefits. This underscores a critical evolution for personalized prescription: clinicians should prioritize extending duration beyond the standard 150-minute weekly benchmark to target sessions of at least 60 minutes. This approach is essential to surmount physiological resistance to lipid adaptation, ensuring the exercise dose is sufficient to transition from simple weight maintenance to active metabolic therapeutic intervention.

Mechanistically, the observed metabolic improvements are intrinsically linked to the optimization of substrate utilization at FATmax intensity. By maximizing the rate of fatty acid oxidation, FATmax training effectively depletes the pool of bioactive lipid intermediates—specifically intramyocellular lipids (IMCL) and diacylglycerol (DAG)—which are potent inhibitors of the insulin signaling cascade (e.g., via IRS-1 phosphorylation) [87, 88]. Beyond acute substrate clearance, training in this zone provides a critical stimulus for mitochondrial biogenesis, characterized by the upregulation of key oxidative enzymes such as citrate synthase (CS) and β-hydroxyacyl-CoA dehydrogenase (β-HAD) [89]. This mitochondrial adaptation enhances the muscle’s “metabolic flexibility” [79, 90, 91], effectively alleviating the lipid-induced blockade on glucose uptake [92] and facilitating greater insulin-stimulated GLUT4 translocation at the sarcolemma [87, 93].

In conclusion, the metabolic implications of FATmax training appear to involve more than just the immediate physiological load. Our analysis suggests that these benefits likely stem from a synergistic interaction among exercise intensity, nutritional status, and individual characteristics. Therefore, by aligning broad clinical guidelines with personalized adjustments for intensity and duration, FATmax training may offer a viable strategy to enhance systemic metabolic flexibility and support general metabolic health in individuals with overweight or obesity.

#### Glucose metabolic capacity

Restoring glucose homeostasis remains a pivotal objective of exercise interventions targeting metabolic health in individuals with overweight or obesity. The present meta-analysis demonstrates that FATmax training significantly ameliorates fasting plasma glucose (FPG), fasting insulin (FINS), and HOMA-IR in this population. Collectively, these findings indicate that FATmax training provides a potent physiological stimulus for enhancing insulin sensitivity and optimizing glycemic control.

Mechanistically, the glucose-lowering effects of FATmax training are inextricably coupled with the regulation of lipid metabolism, a relationship consistent with the classic “glucose-fatty acid cycle” (Randle cycle) [94]. In individuals with obesity, the accumulation of ectopic lipids (such as IMCL) typically creates a state of “metabolic inflexibility”, where muscle cells fail to switch between fuel sources efficiently [91, 95]. FATmax training addresses this by maximizing the oxidation of fatty acids during exercise, which effectively depletes the reservoir of lipid metabolites that interfere with glucose handling [96]. By reducing the “lipid congestion” inside the muscle cell, FATmax re-sensitizes the insulin signaling pathway (specifically the PI3K/Akt axis), thereby restoring the efficiency of insulin-stimulated GLUT4 translocation and glucose uptake [93, 97, 98].

However, the magnitude of these glycemic adaptations appears to be modulated by changes in body composition. While current evidence supports the general efficacy of FATmax training, variations across studies suggest that weight loss acts as a critical mediator. For instance, Mora-Rodriguez et al. reported that in individuals with metabolic syndrome, significant improvements in insulin sensitivity were observed only when exercise-induced body weight reduction exceeded 3% [99]. This implies that while FATmax intensity optimizes substrate utilization, the full restoration of insulin sensitivity relies partly on a concomitant reduction in adiposity. Consequently, the regulatory effects of FATmax on glucose metabolism are likely influenced by individual characteristics, including baseline metabolic state, body fat distribution, and aerobic fitness [100].

From a clinical prescription perspective, our findings support the volume recommendations of the American College of Sports Medicine (ACSM) and the American Diabetes Association (ADA), which suggest 150–300 minutes of moderate-to-vigorous aerobic exercise per week [86, 101, 102]. However, our results argue for a more nuanced definition of “moderate” intensity. Recent evidence suggests that the heart rate corresponding to FATmax should be tailored to body composition. Individuals with body fat percentages above 35% may achieve maximal benefits at 61–66% of HRpeak, whereas those with body fat below 35% may optimize fat oxidation and glycemic stability at 57–64% of HRpeak [103].

To maximize the efficacy of these prescriptions, procedural optimization remains critical, particularly concerning warm-up protocols and nutritional timing. Borghouts et al. demonstrated that an active warm-up enhances glucose metabolism by increasing skeletal muscle temperature and blood flow, thereby facilitating the earlier activation of glucose uptake pathways [104]. Therefore, incorporating standardized warm-up routines serves not only to reduce injury risk but also to prime the metabolic environment for efficient glucose regulation.

Furthermore, the interaction between exercise and nutritional status requires careful consideration. While acute aerobic exercise in a fasted state may transiently elevate circulating glucose and insulin due to metabolic stress [105], chronic training in the fasted state appears to drive superior adaptations. In a 6-week intervention involving a hyper-caloric fat-rich diet, Van Proeyen et al. demonstrated that long-term endurance training performed in the fasted state was more effective than training in the fed state at preserving glucose tolerance. Crucially, this chronic adaptation was underpinned by a significant upregulation of skeletal muscle GLUT4 protein content (+ 28%) and enhanced AMPK phosphorylation (+ 25%), adaptations that were blunted when carbohydrate was ingested before and during exercise [106]. These findings imply that although shortterm fasting may impose transient stress, long-term FATmax training performed in a fasted state can induce beneficial adaptations that enhance metabolic flexibility through improved lipid-glucose crosstalk.

In summary, FATmax training represents a physiologically grounded approach to mitigating insulin resistance by aligning exercise stress with maximal lipid oxidation rates. Nevertheless, interpretations of these findings warrant a prudent perspective. We explicitly state that while the current evidence points towards metabolic benefits, the estimate of the effect size may change with future highquality research. Therefore, the successful clinical application of FATmax relies not merely on the intensity prescription itself but requires a holistic implementation involving precise monitoring, optimized preparation, and supportive nutritional strategies to effectively restore glucose homeostasis.

### Limitations of the study

First, the inclusion criteria were restricted to eligible studies published in English and Chinese, which may have led to the omission of relevant data published in other languages, thereby introducing potential language and publication bias. Although comprehensive searches across major databases were conducted to minimize this risk, the complete elimination of such bias cannot be guaranteed. Second, the distribution of sample sizes across participant characteristics and subgroups was uneven. In particular, male participants accounted for a relatively small proportion of the total sample (193 of 638 participants). This imbalance may have reduced the representativeness and statistical robustness of subgroup analyses, potentially limiting the strength of inferences specific to gender. Third, methodological heterogeneity regarding FATmax determination restricts the precise comparability of intervention effects across studies. The included studies relied on varying assessment protocols (e.g., differences in stage duration and load increments) and distinct mathematical models to identify maximal fat oxidation rates. This lack of standardization introduces potential measurement confounding and variability in the prescribed training intensities, which may obscure the precise magnitude of the FATmax training effect. Finally, the overall number of studies investigating FATmax training in populations with overweight or obesity remains limited, with a geographically concentrated distribution primarily within a few countries, which may restrict the generalizability of our findings. Nevertheless, while sensitivity analyses demonstrated consistent overall trends and stable effect estimates, given the limited sample sizes and statistical power in certain subgroups, the results should be interpreted with appropriate caution.

### Future Research Directions

Based on the identified limitations and the novel findings of this meta-analysis, several critical avenues emerge for future investigation to refine clinical prescriptions. First, future research should prioritize Randomized Controlled Trials (RCTs) designed to explicitly validate the personalized thresholds identified in our interaction analyses. Specifically, experimental studies are needed to compare the differential efficacy of session durations (e.g., ≥ 60 min vs. < 60 min) in individuals with specific phenotypes, such as those with low baseline HDL-C (≤ 1.36 mmol/L), to confirm the causal validity of these regression-derived cutoffs. Second, investigating the synergy between FATmax training and specific nutritional strategies warrants rigorous examination. Future research should explore how FATmax training interacts with interventions such as time-restricted eating (TRE) or distinct macronutrient manipulations to elucidate potential additive effects on metabolic flexibility and substrate oxidation efficiency. Third, to address the geographical and demographic concentration of current data, future investigations must expand to more diverse populations. It is critical to validate these findings across different ethnic groups and in individuals with severe obesity (Class III) to ensure the global generalizability and safety of these precision exercise prescriptions. Fourth, to mitigate methodological heterogeneity and enhance reproducibility, strict adherence to standardized reporting is paramount. We strongly recommend that future investigations explicitly detail the incremental test protocol (including stage duration and load increments), the specifications of gas analysis equipment, and the specific mathematical algorithm utilized for FATmax determination. Such transparency is a prerequisite for high-quality evidence synthesis and the precise translation of research into clinical practice.

## CONCLUSIONS

FATmax training elicits significant metabolic benefits in individuals with overweight or obesity, specifically by improving triglycerides (TG), high-density lipoprotein cholesterol (HDL-C), total cholesterol (TC), fasting plasma glucose (FPG), insulin resistance (HOMA-IR), and fasting insulin (FINS), although changes in low-density lipoprotein cholesterol (LDL-C) are less pronounced. The magnitude of these adaptations is influenced by several moderators: male participants, weight-bearing modalities (e.g., running), and protocols incorporating warm-up sessions generally exhibit superior metabolic enhancements. Furthermore, combining FATmax training with dietary restriction yields additional benefits for HDL-C, though not for FPG. Crucially, our combined subgroup analysis provides precise, actionable guidance for optimizing lipid management: for individuals with lower baseline HDL-C (≤ 1.36 mmol/L), training efficacy is theoretically maximized when session duration exceeds 60 minutes or exercise intensity is maintained above 42.2% $\dot{\text{V}}$O_2max_; conversely, for those with higher baseline HDL-C (> 1.36 mmol/L), extending session duration beyond 60 minutes appears necessary to elicit significant benefits. From a research perspective, while FATmax proves to be an effective metabolic intervention, methodological inconsistencies remain a notable limitation. Future studies must prioritize the adoption of standardized FATmax determination protocols and expand investigations to more diverse populations to strengthen the generalizability of these conclusions and further elucidate the underlying physiological mechanisms.

## Supplementary Material

The impact of maximal fat oxidation intensity exercise on glucose and lipid metabolism in individuals with overweight or obesity: A systematic review and meta-analysis

## Data Availability

The datasets generated and/or analyzed during the current study are available from the corresponding author upon reasonable request. Access to the data will be granted for academic and non-commercial purposes, provided that such sharing complies with ethical standards, participant confidentiality requirements, and institutional data-sharing policies. Additional materials, including analytic code and supplementary documents, can also be made available upon request where applicable.

## Declarations

This study does not involve human participants. All procedures followed in the research comply with the ethical standards of the institution and/or national research committees, as well as the Declaration of Helsinki (1964) and its later amendments or similar ethical guidelines.

## References

[1] World Health Organization. Obesity and overweight. World Health Organization. [updated 2025, cited 2026 January 1]. Available from: https://www.who.int/news-room/fact-sheets/detail/obesity-and-overweight.

[2] Albuquerque D, Nóbrega C, Manco L, Padez C. The contribution of genetics and environment to obesity. Br Med Bull. 2017; 123(1):159–173. doi: 10.1093/bmb/ldx022. 28910990

[3] Finkelstein EA, Khavjou OA, Thompson H, et al. Obesity and severe obesity forecasts through 2030. Am J Prev Med. 2012; 42(6):563–570. doi: 10.1016/j.amepre.2011.10.026. 22608371

[4] González-Muniesa P, Mártinez-González MA, Hu FB, et al. Obesity. Nat Rev Dis Primers. 2017; 3:17034. doi: 10.1038/nrdp.2017.34. 28617414

[5] Haslam DW, James WP. Obesity. Lancet. 2005; 366(9492):1197–1209. doi: 10.1016/S0140-6736(05)67483-1. 16198769

[6] Myers J, Kokkinos P, Nyelin E. Physical Activity, Cardiorespiratory Fitness, and the Metabolic Syndrome. Nutrients. 2019; 11(7):1652. doi: 10.3390/nu11071652. 31331009 PMC6683051

[7] Hawwash NK, Sperrin M, Martin GP, et al. Overweight-years and cancer risk: A prospective study of the association and comparison of predictive performance with body mass index (Atherosclerosis Risk in Communities Study). Int J Cancer. 2024; 154(9):1556–1568. doi: 10.1002/ijc.34821. 38143298 PMC7615716

[8] GBD 2021 Diabetes Collaborators. Global, regional, and national burden of diabetes from 1990 to 2021, with projections of prevalence to 2050: a systematic analysis for the Global Burden of Disease Study 2021. Lancet. 2023; 402(10397):203–234. doi: 10.1016/S0140-6736(23)01301-6. 37356446 PMC10364581

[9] Tutor AW, Lavie CJ, Kachur S, Milani RV, Ventura HO. Updates on obesity and the obesity paradox in cardiovascular diseases. Prog Cardiovasc Dis. 2023; 78:2–10. doi: 10.1016/j.pcad.2022.11.013. 36481212

[10] Shaw K, Gennat H, O’Rourke P, Del Mar C. Exercise for overweight or obesity. Cochrane Database Syst Rev. 2006; 2006(4):CD003817. doi: 10.1002/14651858.CD003817.pub3. 17054187 PMC9017288

[11] Tan SJ, Guo Z, Cao LQ. Study on exercise prescription for weight loss at maximal fat oxidation intensity in obese boys aged 9–10 years. J Sports Sci (China). 2016; 36(9):36–39, 53. doi: 10.16469/j.css.201609005.

[12] Jeukendrup A, Achten J. Fatmax: a new concept to optimize fat oxidation during exercise? Eur J Sport Sci. 2001; 1(5):1–5. doi: 10.1080/17461390100071507.

[13] Brun JF, Myzia J, Varlet-Marie E, Raynaud de Mauverger E, Mercier J. Beyond the Calorie Paradigm: Taking into Account in Practice the Balance of Fat and Carbohydrate Oxidation during Exercise? Nutrients. 2022; 14(8):1605. doi: 10.3390/nu14081605. 35458167 PMC9027421

[14] Hernández-Lepe MA, Hernández-Ontiveros DA, Chávez-Guevara IA, et al. Impact of Exercise Training at Maximal Fat Oxidation Intensity on Metabolic and Epigenetic Parameters in Patients with Overweight and Obesity: Study Protocol of a Randomized Controlled Trial. J Funct Morphol Kinesiol. 2024; 9(4):214. doi: 10.3390/jfmk9040214. 39584867 PMC11587150

[15] Cao L, Jiang Y, Li Q, Wang J, Tan S. Exercise Training at Maximal Fat Oxidation Intensity for Overweight or Obese Older Women: A Randomized Study. J Sports Sci Med. 2019; 18(3):413–418. 31427862 PMC6683615

[16] Jiang Y, Tan S, Wang Z, Guo Z, Li Q, Wang J. Aerobic exercise training at maximal fat oxidation intensity improves body composition, glycemic control, and physical capacity in older people with type 2 diabetes. J Exerc Sci Fit. 2020; 18(1):7–13. doi: 10.1016/j.jesf.2019.08.003. 31641362 PMC6796612

[17] Romain AJ, Carayol M, Desplan M, et al. Physical activity targeted at maximal lipid oxidation: a meta-analysis. J Nutr Metab. 2012; 2012:285395. doi: 10.1155/2012/285395. 22928092 PMC3425832

[18] Chávez-Guevara IA, Urquidez-Romero R, Pérez-León JA, González-Rodríguez E, Moreno-Brito V, Ramos-Jiménez A. Chronic Effect of Fatmax Training on Body Weight, Fat Mass, and Cardiorespiratory Fitness in Obese Subjects: A Meta-Analysis of Randomized Clinical Trials. Int J Environ Res Public Health. 2020; 17(21):7888. doi: 10.3390/ijerph17217888. 33126461 PMC7663534

[19] Lu G. Effects of FATmax exercise on health-related physical fitness and biochemical indicators in obese elderly men. J Jilin Sport Univ. 2023; 39(6):98–103. Article in Chinese. doi: 10.13720/j.cnki.22-1286.2023.06.001.

[20] Page MJ, McKenzie JE, Bossuyt PM, et al. The PRISMA 2020 statement: an updated guideline for reporting systematic reviews. Syst Rev. 2021; 10(1):89. doi: 10.1186/s13643-021-01626-4. 33781348 PMC8008539

[21] Chávez Guevara IA, Amaro-Gahete FJ. Methodological issues related to maximal fat oxidation and FATmax reproducibility: a narrative review. Int J Obes (Lond). 2025; 49(10):1984–1994. doi: 10.1038/s41366-025-01861-y. 40707656

[22] Yin M, Chen Z, Nassis GP, et al. Chronic high-intensity interval training and moderate-intensity continuous training are both effective in increasing maximum fat oxidation during exercise in overweight and obese adults: A meta-analysis. J Exerc Sci Fit. 2023; 21(4):354–365. doi: 10.1016/j.jesf.2023.08.001. 37701124 PMC10494468

[23] McHugh ML. Interrater reliability: the kappa statistic. Biochem Med (Zagreb). 2012; 22(3):276–282. 23092060 PMC3900052

[24] Achten J, Gleeson M, Jeukendrup AE. Determination of the exercise intensity that elicits maximal fat oxidation. Med Sci Sports Exerc. 2002; 34(1):92–97. doi: 10.1097/00005768-200201000-00015. 11782653

[25] Meyer T, Folz C, Rosenberger F, Kindermann W. The reliability of fatmax. Scand J Med Sci Sports. 2009; 19(2):213–221. doi: 10.1111/j.1600-0838.2008.00775.x. 18282220

[26] Drevon D, Fursa SR, Malcolm AL. Intercoder Reliability and Validity of WebPlotDigitizer in Extracting Graphed Data. Behav Modif. 2017; 41(2):323–339. doi: 10.1177/0145445516673998. 27760807

[27] Sterne JA, Hernán MA, Reeves BC, et al. ROBINS-I: a tool for assessing risk of bias in non-randomised studies of interventions. BMJ. 2016; 355:i4919. doi: 10.1136/bmj.i4919. 27733354 PMC5062054

[28] De Morton NA. The PEDro scale is a valid measure of the methodological quality of clinical trials: a demographic study. Aust J Physiother. 2009; 55(2):129–133. doi: 10.1016/s0004-9514(09)70043-1. 19463084

[29] Becker BJ. Synthesizing standardized mean-change measures. Br J Math Stat Psychol. 1988; 41(2):257–278. doi: 10.1111/j.2044-8317.1988.tb00901.x.

[30] Morris SB, DeShon RP. Combining effect size estimates in meta-analysis with repeated measures and independentgroups designs. Psychol Methods. 2002; 7(1):105–125. doi: 10.1037/1082-989x.7.1.105. 11928886

[31] Morris SB. Estimating effect sizes from pretest – posttest – control group designs. Organ Res Methods. 2008; 11(2):364–386. doi: 10.1177/1094428106291059.

[32] Cumpston M, Li T, Page MJ, et al. Updated guidance for trusted systematic reviews: a new edition of the Cochrane Handbook for Systematic Reviews of Interventions. Cochrane Database Syst Rev. 2019; 10(10):ED000142. doi: 10.1002/14651858.ED000142. 31643080 PMC10284251

[33] Follmann D, Elliott P, Suh I, Cutler J. Variance imputation for overviews of clinical trials with continuous response. J Clin Epidemiol. 1992; 45(7):769–773. doi: 10.1016/0895-4356(92)90054-q. 1619456

[34] Wewege M, van den Berg R, Ward RE, Keech A. The effects of high-intensity interval training vs. moderate-intensity continuous training on body composition in overweight and obese adults: a systematic review and meta-analysis. Obes Rev. 2017; 18(6):635–646. doi: 10.1111/obr.12532. 28401638

[35] Hedges LV, Olkin I. Random effects models for effect sizes. In: Statistical Methods for Meta-Analysis. Academic Press; 1985:245–279.

[36] Cohen J. Statistical Power Analysis for the Behavioral Sciences. Routledge; 2013.

[37] Hopkins WG. Improving meta-analyses in sport and exercise science. Sportscience. 2018; 22:11–19.

[38] Ruppar T. Meta-analysis: How to quantify and explain heterogeneity? Eur J Cardiovasc Nurs. 2020; 19(7):646–652. doi: 10.1177/1474515120944014. 32757621

[39] Quintana DS. A guide for calculating study-level statistical power for meta-analyses. Adv Methods Pract Psychol Sci. 2023; 6(1). doi: 10.1177/25152459221147260.

[40] Christ A. Mixed effects models and extensions in ecology with R. J Stat Softw. 2009; 32(1):1–3. doi: 10.18637/jss.v032.b01.

[41] Harrell FE. General aspects of fitting regression models. In: Regression Modeling Strategies. Springer; 2001. doi: 10.1007/978-1-4757-3462-1_2.

[42] Wickham H. ggplot2. Wiley Interdiscip Rev Comput Stat. 2011; 3(2):180–185. doi: 10.1002/wics.147.

[43] Peters JL, Sutton AJ, Jones DR, Abrams KR, Rushton L. Contourenhanced meta-analysis funnel plots help distinguish publication bias from other causes of asymmetry. J Clin Epidemiol. 2008; 61(10):991–996. doi: 10.1016/j.jclinepi.2007.11.010. 18538991

[44] Egger M, Davey Smith G, Schneider M, Minder C. Bias in meta-analysis detected by a simple, graphical test. BMJ. 1997; 315(7109):629–634. doi: 10.1136/bmj.315.7109.629. 9310563 PMC2127453

[45] Fernández-Castilla B, Declercq L, Jamshidi L, Beretvas SN, Onghena P, Van den Noortgate W. Detecting selection bias in meta-analyses with multiple outcomes: a simulation study. J Exp Educ. 2021; 89(1):125–144. doi: 10.1080/00220973.2019.1582470. 32808180

[46] Sterne JA, Sutton AJ, Ioannidis JP, et al. Recommendations for examining and interpreting funnel plot asymmetry in meta-analyses of randomised controlled trials. BMJ. 2011; 343:d4002. doi: 10.1136/bmj.d4002. 21784880

[47] Viechtbauer W, Cheung MW. Outlier and influence diagnostics for meta-analysis. Res Synth Methods. 2010; 1(2):112–125. doi: 10.1002/jrsm.11. 26061377

[48] Cook RD, Weisberg S. Residuals and Influence in Regression. Chapman and Hall; 1982.

[49] Schünemann HJ, Higgins JP, Vist GE, et al. Completing ‘summary of findings’ tables and grading the certainty of the evidence. In: Cochrane Handbook for Systematic Reviews of Interventions. 2019:375–402. doi: 10.1002/9781119536604.ch14.

[50] Ben Ounis O, Elloumi M, Amri M, Zbidi A, Tabka Z, Lac G. Impact of diet, exercise end diet combined with exercise programs on plasma lipoprotein and adiponectin levels in obese girls. J Sports Sci Med. 2008; 7(4):437–445. 24149948 PMC3761909

[51] Ben Ounis O, Elloumi M, Ben Chiekh I, et al. Effects of two-month physicalendurance and diet-restriction programmes on lipid profiles and insulin resistance in obese adolescent boys. Diabetes Metab. 2008; 34(6 Pt 1):595–600. doi: 10.1016/j.diabet.2008.05.011. 18930691

[52] Tan S, Wang X, Wang J. Effects of supervised exercise training at the intensity of maximal fat oxidation in overweight young women. J Exerc Sci Fit. 2012; 10(2):64–69. doi: 10.1016/j.jesf.2012.10.002.

[53] Tan S, Wang J, Cao L, Guo Z, Wang Y. Positive effect of exercise training at maximal fat oxidation intensity on body composition and lipid metabolism in overweight middle-aged women. Clin Physiol Funct Imaging. 2016; 36(3):225–230. doi: 10.1111/cpf.12217. 27072372

[54] Tan S, Du P, Zhao W, Pang J, Wang J. Exercise Training at Maximal Fat Oxidation Intensity for Older Women with Type 2 Diabetes. Int J Sports Med. 2018; 39(5):374–381. doi: 10.1055/a-0573-1509. 29564847

[55] Huang C, Pan M, Zhang L, et al. Effect of exercise at maximal fat oxidation intensity on arterial stiffness in overweight or obese young men. Chin J Sports Med. 2018; 37(1):3–9. Article in Chinese. doi: 10.16038/j.1000-6710.2018.01.001.

[56] Jiang Y, Tan S, Li Q. Research on exercise intervention prescription for middle-aged male patients with metabolic syndrome based on FATmax theory. J Tianjin Univ Sport. 2020; 35(5):545–548, 565. Article in Chinese. doi: 10.13297/j.cnki.issn1005-0000.2020.05.009.

[57] Tan S, Xu D, Cao L, et al. Effect of FATmax exercise intervention on non-alcoholic fatty liver disease in middle-aged women. J Tianjin Univ Sport. 2015; 30(3):185–189. Article in Chinese. doi: 10.13297/j.cnki.issn1005-0000.2015.03.001.

[58] Venables MC, Jeukendrup AE. Endurance training and obesity: effect on substrate metabolism and insulin sensitivity. Med Sci Sports Exerc. 2008; 40(3):495–502. doi: 10.1249/MSS.0b013e31815f256f. 18379212

[59] Lanzi S, Codecasa F, Cornacchia M, et al. Short-term HIIT and Fat max training increase aerobic and metabolic fitness in men with class II and III obesity. Obesity (Silver Spring). 2015; 23(10):1987–1994. doi: 10.1002/oby.21206. 26335027

[60] Kantorowicz M, Szymura J, Szygula Z, Kusmierczyk J, Maciejczyk M, Wiecek M. Nordic Walking at Maximal Fat Oxidation Intensity Decreases Circulating Asprosin and Visceral Obesity in Women With Metabolic Disorders. Front Physiol. 2021; 12:726783. doi: 10.3389/fphys.2021.726783. 34539448 PMC8446531

[61] Mohebbi H, Nourshahi M, Ghasemikaram M, Safarimosavi S. Effects of exercise at individual anaerobic threshold and maximal fat oxidation intensities on plasma levels of nesfatin-1 and metabolic health biomarkers. J Physiol Biochem. 2015; 71(1):79–88. doi: 10.1007/s13105-015-0383-2. 25637303

[62] Besnier F, Lenclume V, Gérardin P, et al. Individualized Exercise Training at Maximal Fat Oxidation Combined with Fruit and Vegetable-Rich Diet in Overweight or Obese Women: The LIPOXmax-Réunion Randomized Controlled Trial. PLoS One. 2015; 10(11):e0139246. Published 2015 Nov 10. doi: 10.1371/journal.pone.0139246. 26555595 PMC4640859

[63] Safarimosavi S, Mohebbi H, Rohani H. High-Intensity Interval vs. Continuous Endurance Training: Preventive Effects on Hormonal Changes and Physiological Adaptations in Prediabetes Patients. J Strength Cond Res. 2021; 35(3):731–738. doi: 10.1519/JSC.0000000000002709. 29939900

[64] Ben Ounis O, Elloumi M, Lac G, et al. Two-month effects of individualized exercise training with or without caloric restriction on plasma adipocytokine levels in obese female adolescents. Ann Endocrinol (Paris). 2009; 70(4):235–241. doi: 10.1016/j.ando.2009.03.003. 19403116

[65] Dumortier M, Brandou F, Perez-Martin A, Fedou C, Mercier J, Brun JF. Low intensity endurance exercise targeted for lipid oxidation improves body composition and insulin sensitivity in patients with the metabolic syndrome. Diabetes Metab. 2003; 29(5):509–518. doi: 10.1016/s1262-3636(07)70065-4. 14631328

[66] Maurie J, Brun JF, Jean E, Romain AJ, Mercier J. Comparaison de deux modalités différentes d’activité physique (SWEET et Lipoxmax) chez des diabétiques de type 2. Sci Sports. 2011; 26(2):92–96. doi: 10.1016/j.scispo.2010.12.002.

[67] Ben Ounis O, Elloumi M, Makni E, et al. Exercise improves the ApoB/ApoA-I ratio, a marker of the metabolic syndrome in obese children. Acta Paediatr. 2010; 99(11):1679–1685. doi: 10.1111/j.1651-2227.2010.01920.x. 20594189

[68] Wang D, Zhang P, Li J. Crossover point and maximal fat oxidation training effects on blood lipid metabolism in young overweight women: a pilot study. Front Physiol. 2023; 14:1190109. doi: 10.3389/fphys.2023.1190109. 37398909 PMC10311904

[69] Elloumi M, Ben Ounis O, Makni E, Van Praagh E, Tabka Z, Lac G. Effect of individualized weight-loss programmes on adiponectin, leptin and resistin levels in obese adolescent boys. Acta Paediatr. 2009; 98(9):1487–1493. doi: 10.1111/j.1651-2227.2009.01365.x. 19489770

[70] Mann S, Beedie C, Jimenez A. Differential effects of aerobic exercise, resistance training and combined exercise modalities on cholesterol and the lipid profile: review, synthesis and recommendations. Sports Med. 2014; 44(2):211–221. doi: 10.1007/s40279-013-0110-5. 24174305 PMC3906547

[71] Kodama S, Tanaka S, Saito K, et al. Effect of aerobic exercise training on serum levels of high-density lipoprotein cholesterol: a meta-analysis. Arch Intern Med. 2007; 167(10):999–1008. doi: 10.1001/archinte.167.10.999. 17533202

[72] Brown MS, Goldstein JL. A receptormediated pathway for cholesterol homeostasis. Science. 1986; 232(4746):34–47. doi: 10.1126/science.3513311. 3513311

[73] Wang Y, Xu D. Effects of aerobic exercise on lipids and lipoproteins. Lipids Health Dis. 2017; 16(1):132. doi: 10.1186/s12944-017-0515-5. 28679436 PMC5498979

[74] Stancu C, Sima A. Statins: mechanism of action and effects. J Cell Mol Med. 2001; 5(4):378–387. doi: 10.1111/j.1582-4934.2001.tb00172.x. 12067471 PMC6740083

[75] Albarrati AM, Alghamdi MSM, Nazer RI, Alkorashy MM, Alshowier N, Gale N. Effectiveness of Low to Moderate Physical Exercise Training on the Level of Low-Density Lipoproteins: A Systematic Review. Biomed Res Int. 2018; 2018:5982980. doi: 10.1155/2018/5982980. 30515408 PMC6236809

[76] Durstine JL, Haskell WL. Effects of exercise training on plasma lipids and lipoproteins. Exerc Sport Sci Rev. 1994; 22:477–521. 7925552

[77] Goldberg L, Elliot DL. The effect of exercise on lipid metabolism in men and women. Sports Med. 1987; 4(5):307–321. doi: 10.2165/00007256-198704050-00001. 3313616

[78] Frandsen J, Poggi AI, Ritz C, Larsen S, Dela F, Helge JW. Peak Fat Oxidation Rate Is Closely Associated With Plasma Free Fatty Acid Concentrations in Women; Similar to Men. Front Physiol. 2021; 12:696261. doi: 10.3389/fphys.2021.696261. 34408659 PMC8364948

[79] Goodpaster BH, Sparks LM. Metabolic Flexibility in Health and Disease. Cell Metab. 2017; 25(5):1027–1036. doi: 10.1016/j.cmet.2017.04.015. 28467922 PMC5513193

[80] Blaak EE. Basic disturbances in skeletal muscle fatty acid metabolism in obesity and type 2 diabetes mellitus. Proc Nutr Soc. 2004; 63(2):323–330. doi: 10.1079/PNS2004361. 15294050

[81] Brun JF, Fedou C, Grubka E, Karafiat M, Varlet-Marie E, Mercier J. Moindre utilisation des lipides à l’exercice chez le diabétique de type 1. Sci Sports. 2008; 23(3–4):198–200. doi: 10.1016/j.scispo.2007.12.016.

[82] Gray SC, Devito G, Nimmo MA. Effect of active warm-up on metabolism prior to and during intense dynamic exercise. Med Sci Sports Exerc. 2002; 34(12):2091–2096. doi: 10.1097/00005768-200212000-00034. 12471321

[83] Achten J, Venables MC, Jeukendrup AE. Fat oxidation rates are higher during running compared with cycling over a wide range of intensities. Metabolism. 2003; 52(6):747–752. doi: 10.1016/s0026-0495(03)00068-4. 12800102

[84] Thomas TR, Feiock CW, Araujo J. Metabolic responses associated with four modes of prolonged exercise. J Sports Med Phys Fitness. 1989; 29(1):77–82. 2770272

[85] Garber CE, Blissmer B, Deschenes MR, et al. American College of Sports Medicine position stand. Quantity and quality of exercise for developing and maintaining cardiorespiratory, musculoskeletal, and neuromotor fitness in apparently healthy adults: guidance for prescribing exercise. Med Sci Sports Exerc. 2011; 43(7):1334–1359. doi: 10.1249/MSS.0b013e318213fefb. 21694556

[86] Jakicic JM, Apovian CM, Barr-Anderson DJ, et al. Physical Activity and Excess Body Weight and Adiposity for Adults. American College of Sports Medicine Consensus Statement. Med Sci Sports Exerc. 2024; 56(10):2076–2091. doi: 10.1249/MSS.0000000000003520. 39277776

[87] Samuel VT, Shulman GI. Mechanisms for insulin resistance: common threads and missing links. Cell. 2012; 148(5):852–871. doi: 10.1016/j.cell.2012.02.017. 22385956 PMC3294420

[88] Itani SI, Ruderman NB, Schmieder F, Boden G. Lipid-induced insulin resistance in human muscle is associated with changes in diacylglycerol, protein kinase C, and IkappaB-alpha. Diabetes. 2002; 51(7):2005–2011. doi: 10.2337/diabetes.51.7.2005. 12086926

[89] Holloszy JO, Coyle EF. Adaptations of skeletal muscle to endurance exercise and their metabolic consequences. J Appl Physiol Respir Environ Exerc Physiol. 1984; 56(4):831–838. doi: 10.1152/jappl.1984.56.4.831. 6373687

[90] San-Millán I. The Key Role of Mitochondrial Function in Health and Disease. Antioxidants (Basel). 2023; 12(4):782. doi: 10.3390/antiox12040782. 37107158 PMC10135185

[91] Kelley DE, Mandarino LJ. Fuel selection in human skeletal muscle in insulin resistance: a reexamination. Diabetes. 2000; 49(5):677–683. doi: 10.2337/diabetes.49.5.677. 10905472

[92] Mambrini SP, Grillo A, Colosimo S, et al. Diet and physical exercise as key players to tackle MASLD through improvement of insulin resistance and metabolic flexibility. Front Nutr. 2024; 11:1426551. Published 2024 Aug 20. doi: 10.3389/fnut.2024.1426551. 39229589 PMC11370663

[93] Richter EA, Hargreaves M. Exercise, GLUT4, and skeletal muscle glucose uptake. Physiol Rev. 2013; 93(3):993–1017. doi: 10.1152/physrev.00038.2012. 23899560

[94] Randle PJ, Garland PB, Hales CN, Newsholme EA. The glucose fatty-acid cycle. Its role in insulin sensitivity and the metabolic disturbances of diabetes mellitus. Lancet. 1963; 1(7285):785–789. doi: 10.1016/s0140-6736(63)91500-9. 13990765

[95] Chu L, Morrison KM, Riddell MC, Raha S, Timmons BW. Metabolic Flexibility during Exercise in Children with Obesity and Matched Controls. Med Sci Sports Exerc. 2021; 53(1):159–164. doi: 10.1249/MSS.0000000000002428. 32520873

[96] Schenk S, Horowitz JF. Acute exercise increases triglyceride synthesis in skeletal muscle and prevents fatty acid-induced insulin resistance. J Clin Invest. 2007; 117(6):1690–1698. doi: 10.1172/JCI30566. 17510709 PMC1866251

[97] Sylow L, Kleinert M, Richter EA, Jensen TE. Exercise-stimulated glucose uptake – regulation and implications for glycaemic control. Nat Rev Endocrinol. 2017; 13(3):133–148. doi: 10.1038/nrendo.2016.162. 27739515

[98] O’Gorman DJ, Karlsson HK, McQuaid S, et al. Exercise training increases insulin-stimulated glucose disposal and GLUT4 (SLC2A4) protein content in patients with type 2 diabetes. Diabetologia. 2006; 49(12):2983–2992. doi: 10.1007/s00125-006-0457-3. 17019595

[99] Mora-Rodriguez R, Ortega JF, Ramirez-Jimenez M, Moreno-Cabañas A, Morales-Palomo F. Insulin sensitivity improvement with exercise training is mediated by body weight loss in subjects with metabolic syndrome. Diabetes Metab. 2020; 46(3):210–218. doi: 10.1016/j.diabet.2019.05.004. 31158474

[100] Böhm A, Weigert C, Staiger H, Häring HU. Exercise and diabetes: relevance and causes for response variability. Endocrine. 2016; 51(3):390–401. doi: 10.1007/s12020-015-0792-6. 26643313 PMC4762932

[101] Colberg SR, Sigal RJ, Yardley JE, et al. Physical Activity/Exercise and Diabetes: A Position Statement of the American Diabetes Association. Diabetes Care. 2016; 39(11):2065–2079. doi: 10.2337/dc16-1728. 27926890 PMC6908414

[102] Kanaley JA, Colberg SR, Corcoran MH, et al. Exercise/Physical Activity in Individuals with Type 2 Diabetes: A Consensus Statement from the American College of Sports Medicine. Med Sci Sports Exerc. 2022; 54(2):353–368. doi: 10.1249/MSS.0000000000002800. 35029593 PMC8802999

[103] Chávez-Guevara IA, Amaro-Gahete FJ, Ramos-Jiménez A, Brun JF. Toward Exercise Guidelines for Optimizing Fat Oxidation During Exercise in Obesity: A Systematic Review and Meta-Regression. Sports Med. 2023; 53(12):2399–2416. doi: 10.1007/s40279-023-01897-y. 37584843

[104] Borghouts LB, Keizer HA. Exercise and insulin sensitivity: a review. Int J Sports Med. 2000; 21(1):1–12. doi: 10.1055/s-2000-8847. 10683091

[105] Kazeminasab F, Rafiee P, Miraghajani M, Santos HO, Symonds ME, Rosenkranz SK. The effects of acute bouts of exercise in fasted vs. fed states on glucose and lipid metabolism in healthy adults: A systematic review and meta-analysis of randomized clinical trials. Clin Nutr ESPEN. 2025; 66:320–331. doi: 10.1016/j.clnesp.2025.02.002. 39921164

[106] Van Proeyen K, Szlufcik K, Nielens H, et al. Training in the fasted state improves glucose tolerance during fat-rich diet. J Physiol. 2010; 588(Pt 21):4289–4302. doi: 10.1113/jphysiol.2010.196493. 20837645 PMC3002457

