# Supplementary material for: The impact of maximal fat oxidation intensity exercise on glucose and lipid metabolism in individuals with overweight or obesity: A systematic review and meta-analysis
Source: Biol Sport. 2026 Apr 13;43:1193–210. doi: 10.5114/biolsport.2026.159565 (PMC13343287; doi:10.5114/biolsport.2026.159565)
Supplement: The impact of maximal fat oxidation intensity exercise on glucose and lipid metabolism in individuals with overweight or obesity: A systematic review and meta-analysis [file JBS-43-57604-s1.pdf]

SUPPLEMENTARY MATERIAL
GRAPHICAL ABSTRACT

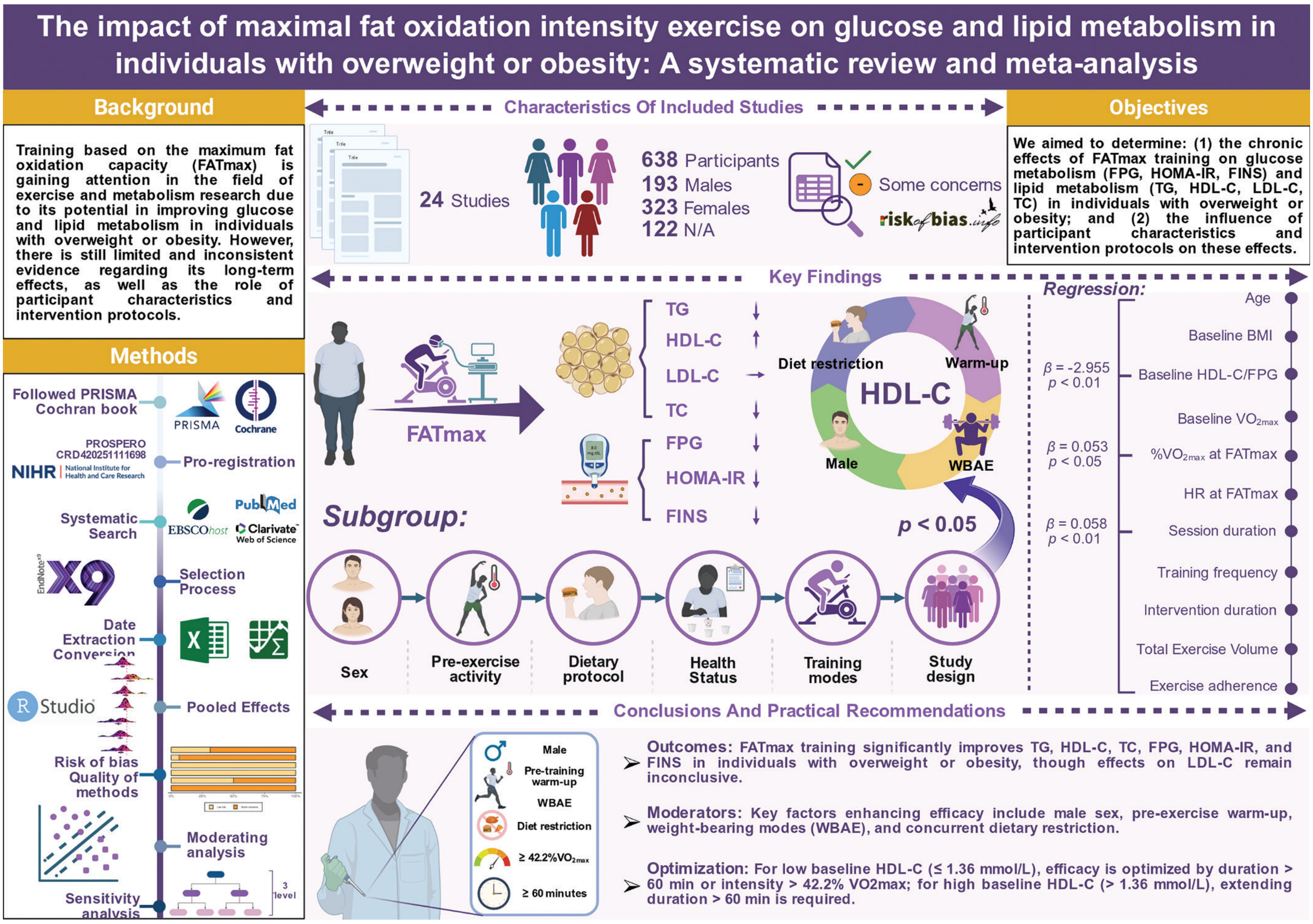

SUPPLEMENTARY FIG. 1. Abstract

SUPPLEMENTARY TABLE 1. PRISMA 2020 Checklist

Section and Topic	Item #	Checklist item	Location where item is reported
**TITLE**			
Title	1	Identify the report as a systematic review.	Pages 1 (manuscript)
**ABSTRACT**			
Abstract	2	See the PRISMA 2020 for Abstracts checklist.	Pages 1 (manuscript)
**INTRODUCTION**			
Rationale	3	Describe the rationale for the review in the context of existing knowledge.	Pages 1–2 (manuscript)
Objectives	4	Provide an explicit statement of the objective(s) or question(s) the review addresses.	Pages 1–2 (manuscript)
**METHODS**			
Eligibility criteria	5	Specify the inclusion and exclusion criteria for the review and how studies were grouped for the syntheses.	Pages 2–3 (manuscript)
Information sources	6	Specify all databases, registers, websites, organisations, reference lists and other sources searched or consulted to identify studies. Specify the date when each source was last searched or consulted.	Pages 3 (manuscript)
Search strategy	7	Present the full search strategies for all databases, registers and websites, including any filters and limits used.	Pages 25-27 (supplementary material)
Selection process	8	Specify the methods used to decide whether a study met the inclusion criteria of the review, including how many reviewers screened each record and each report retrieved, whether they worked independently, and if applicable, details of automation tools used in the process.	Pages 3 (manuscript)
Data collection process	9	Specify the methods used to collect data from reports, including how many reviewers collected data from each report, whether they worked independently, any processes for obtaining or confirming data from study investigators, and if applicable, details of automation tools used in the process.	Pages 3 (manuscript)
Data items	10a	List and define all outcomes for which data were sought. Specify whether all results that were compatible with each outcome domain in each study were sought (e.g. for all measures, time points, analyses), and if not, the methods used to decide which results to collect.	Pages 3 (manuscript)
	10b	List and define all other variables for which data were sought (e.g. participant and intervention characteristics, funding sources). Describe any assumptions made about any missing or unclear information.	Pages 25-27 (supplementary material)
Study risk of bias assessment	11	Specify the methods used to assess risk of bias in the included studies, including details of the tool(s) used, how many reviewers assessed each study and whether they worked independently, and if applicable, details of automation tools used in the process.	Pages 4 (manuscript)
Effect measures	12	Specify for each outcome the effect measure(s) (e.g. risk ratio, mean difference) used in the synthesis or presentation of results.	Pages 4 (manuscript)
Synthesis methods	13a	Describe the processes used to decide which studies were eligible for each synthesis (e.g. tabulating the study intervention characteristics and comparing against the planned groups for each synthesis (item #5)).	Pages 3 (manuscript)
	13b	Describe any methods required to prepare the data for presentation or synthesis, such as handling of missing summary statistics, or data conversions.	Pages 4 (manuscript)
	13c	Describe any methods used to tabulate or visually display results of individual studies and syntheses.	Pages 4 (manuscript)

SUPPLEMENTARY TABLE 1. Continue.

Section and Topic	Item #	Checklist item	Location where item is reported
	13d	Describe any methods used to synthesize results and provide a rationale for the choice(s). If meta-analysis was performed, describe the model(s), method(s) to identify the presence and extent of statistical heterogeneity, and software package(s) used.	Pages 4 (manuscript)
	13e	Describe any methods used to explore possible causes of heterogeneity among study results (e.g. subgroup analysis, meta-regression).	Pages 4 (manuscript)
	13f	Describe any sensitivity analyses conducted to assess robustness of the synthesized results.	Pages 5 (manuscript)
Reporting bias assessment	14	Describe any methods used to assess risk of bias due to missing results in a synthesis (arising from reporting biases).	Pages 4 (manuscript)
Certainty assessment	15	Describe any methods used to assess certainty (or confidence) in the body of evidence for an outcome.	Pages 5 (manuscript)
**RESULTS**			
Study selection	16a	Describe the results of the search and selection process, from the number of records identified in the search to the number of studies included in the review, ideally using a flow diagram.	Pages 5 (manuscript)
	16b	Cite studies that might appear to meet the inclusion criteria, but which were excluded, and explain why they were excluded.	Pages 3 (manuscript)
Study characteristics	17	Cite each included study and present its characteristics.	Pages 25-27 (supplementary material)
Risk of bias in studies	18	Present assessments of risk of bias for each included study.	Page 39 (supplementary material)
Results of individual studies	19	For all outcomes, present, for each study: (a) summary statistics for each group (where appropriate) and (b) an effect estimate and its precision (e.g. confidence/credible interval), ideally using structured tables or plots.	Pages 6–7 (manuscript)
Results of syntheses	20a	For each synthesis, briefly summarise the characteristics and risk of bias among contributing studies.	Pages 9–11 (manuscript)
	20b	Present results of all statistical syntheses conducted. If meta-analysis was done, present for each the summary estimate and its precision (e.g. confidence/credible interval) and measures of statistical heterogeneity. If comparing groups, describe the direction of the effect.	Pages 6–10 (manuscript)
	20c	Present results of all investigations of possible causes of heterogeneity among study results.	Pages 6–11 (manuscript)
	20d	Present results of all sensitivity analyses conducted to assess the robustness of the synthesized results.	Pages 10 (manuscript)
Reporting biases	21	Present assessments of risk of bias due to missing results (arising from reporting biases) for each synthesis assessed.	Pages 9–10 (manuscript)
Certainty of evidence	22	Present assessments of certainty (or confidence) in the body of evidence for each outcome assessed.	Pages 6–7 (manuscript)
**DISCUSSION**			
Discussion	23a	Provide a general interpretation of the results in the context of other evidence.	Pages 11–13 (manuscript)
	23b	Discuss any limitations of the evidence included in the review.	Pages 13–14 (manuscript)
	23c	Discuss any limitations of the review processes used.	Pages 13–14 (manuscript)
	23d	Discuss implications of the results for practice, policy, and future research.	Pages 14 (manuscript)

SUPPLEMENTARY TABLE 1. Continue.

Section and Topic	Item #	Checklist item	Location where item is reported
**OTHER INFORMATION**			
Registration and protocol	24a	Provide registration information for the review, including register name and registration number, or state that the review was not registered.	Pages 2 (manuscript)
	24b	Indicate where the review protocol can be accessed, or state that a protocol was not prepared.	n/a
	24c	Describe and explain any amendments to information provided at registration or in the protocol.	n/a
Support	25	Describe sources of financial or non-financial support for the review, and the role of the funders or sponsors in the review.	Pages 14 (manuscript)
Competing interests	26	Declare any competing interests of review authors.	Pages 14 (manuscript)
Availability of data, code and other materials	27	Report which of the following are publicly available and where they can be found: template data collection forms; data extracted from included studies; data used for all analyses; analytic code; any other materials used in the review.	Pages 14–15 (manuscript)

From: Page MJ, McKenzie JE, Bossuyt PM, Boutron I, Hoffmann TC, Mulrow CD, et al. The PRISMA 2020 statement: an updated guideline for reporting systematic reviews. BMJ 2021;372:n71. doi: 10.1136/bmj.n71. This work is licensed under CC BY 4.0. To view a copy of this license, visit https://creativecommons.org/licenses/by/4.0/

SUPPLEMENTARY TABLE 2. First Search

Data	Query	Results
**Pubmed**	((FATmax [Title/Abstract]) OR (LIPOXmax [Title/Abstract])) OR (maximal fat oxidation) OR (fat metabolism) AND ((training [Title/Abstract]) OR (exercise [Title/Abstract])) OR (physical activity) AND ((obesity [Title/Abstract]) OR (overweight [Title/Abstract]) OR (obese [Title/Abstract])) AND ((blood glucose [Title/Abstract]) OR (blood lipids [Title/Abstract])) OR (lipid metabolism) OR (glucose metabolism) OR (lipid profile) AND ((randomized controlled trial [Title/Abstract]) OR (controlled clinical trial [Title/Abstract]) OR (RCT [Title/Abstract]))	**153**
**Web of Science**	TS=(FATmax) OR TS=(LIPOXmax) OR ALL=(FATOXmax) OR ALL=(maximal fat oxidation) OR ALL=(fat metabolism) AND (TS=(training) OR TS=(exercise) OR ALL=(physical activity) AND ((TS=(obesity) OR TS=(overweight)) OR ALL=(obese) AND (TS=(blood glucose) OR TS=(blood lipids)) OR ALL=(lipid metabolism) AND TS=(randomized controlled trial) OR TS=(controlled clinical trial)) OR TS=(RCT)	**93**
**EBSCO**	SU FATmax OR SU LIPOXmax OR FATOXmax OR maximal fat oxidation OR fat metabolism AND SU training AND SU obesity OR SU overweight OR obese AND SU blood glucose OR SU blood lipids OR lipid metabolism AND SU randomized controlled trial OR SU controlled clinical trial OR SU RCT	**322**
**ICTRP**	(FATmax OR LIPOXmax OR FATOXmax OR maximal fat oxidation OR fat metabolism) AND (training OR exercise OR physical activity) AND (obesity OR overweight OR obese) AND (blood glucose OR blood lipids OR lipid metabolism) AND (randomized controlled trial OR controlled clinical trial RCT)	**183**
**Cochrane**	(FATmax):ti,ab,kw OR (LIPOXmax):ti,ab,kw OR (maximal fat oxidation) OR (fat metabolism) (Word variations have been searched) AND (training):ti,ab,kw OR (exercise):ti,ab,kw (Word variations have been searched) AND (obesity):ti,ab,kw OR (overweight):ti,ab,kw OR (obese) (Word variations have been searched) AND (blood glucose):ti,ab,kw OR (blood lipids):ti,ab,kw OR (lipid metabolism) AND (randomized controlled trial):ti,ab,kw OR (controlled clinical trial):ti,ab,kw OR (RCT):ti,ab,kw	**678**
**Embase**	(FATmax OR LIPOXmax OR FATOXmax OR maximal fat oxidation OR fat metabolism) AND (training OR exercise OR physical activity) AND (obesity OR overweight OR obese) AND (blood glucose OR blood lipids OR lipid metabolism) AND (randomized controlled trial OR controlled clinical trial RCT)	**48**
**CNKI**	(主题：FATmax) OR (主题：LIPOXmax) OR (主题：最大脂肪氧化强度) AND (主题：运动) OR (主题：训练) AND (主题：随机) OR (主题：对照) OR (主题：试验)	**99**

Note: ICTRP = International Clinical Trials Registry Platform; CNKI = Chinese National Knowledge Infrastructure.

SUPPLEMENTARY TABLE 3. Updated Search

Data	Query	Results
**Pubmed**	((FATmax [Title/Abstract]) OR (LIPOXmax [Title/Abstract])) OR (maximal fat oxidation) OR (fat metabolism) AND ((training [Title/Abstract]) OR (exercise [Title/Abstract])) OR (physical activity) AND ((obesity [Title/Abstract]) OR (overweight [Title/Abstract]) OR (obese [Title/Abstract])) AND ((blood glucose [Title/Abstract]) OR (blood lipids [Title/Abstract])) OR (lipid metabolism) OR (glucose metabolism) OR (lipid profile) AND ((randomized controlled trial [Title/Abstract]) OR (controlled clinical trial [Title/Abstract]) OR (RCT [Title/Abstract]))	**11**
**Web of Science**	TS=(FATmax) OR TS=(LIPOXmax) OR ALL=(FATOXmax) OR ALL=(maximal fat oxidation) OR ALL=(fat metabolism) AND (TS=(training) OR TS=(exercise) OR ALL=(physical activity) AND ((TS=(obesity) OR TS=(overweight)) OR ALL=(obese) AND (TS=(blood glucose) OR TS=(blood lipids)) OR ALL=(lipid metabolism) AND TS=(randomized controlled trial) OR TS=(controlled clinical trial)) OR TS=(RCT)	**6**
**EBSCO**	SU FATmax OR SU LIPOXmax OR FATOXmax OR maximal fat oxidation OR fat metabolism AND SU training AND SU obesity OR SU overweight OR obese AND SU blood glucose OR SU blood lipids OR lipid metabolism AND SU randomized controlled trial OR SU controlled clinical trial OR SU RCT	**10**
**ICTRP**	(FATmax OR LIPOXmax OR FATOXmax OR maximal fat oxidation OR fat metabolism) AND (training OR exercise OR physical activity) AND (obesity OR overweight OR obese) AND (blood glucose OR blood lipids OR lipid metabolism) AND (randomized controlled trial OR controlled clinical trial RCT)	**4**
**Cochrane**	(FATmax):ti,ab,kw OR (LIPOXmax):ti,ab,kw OR (maximal fat oxidation) OR (fat metabolism) (Word variations have been searched) AND (training):ti,ab,kw OR (exercise):ti,ab,kw (Word variations have been searched) AND (obesity):ti,ab,kw OR (overweight):ti,ab,kw OR (obese) (Word variations have been searched) AND (blood glucose):ti,ab,kw OR (blood lipids):ti,ab,kw OR (lipid metabolism) AND (randomized controlled trial):ti,ab,kw OR (controlled clinical trial):ti,ab,kw OR (RCT):ti,ab,kw	**18**
**Embase**	(FATmax OR LIPOXmax OR FATOXmax OR maximal fat oxidation OR fat metabolism) AND (training OR exercise OR physical activity) AND (obesity OR overweight OR obese) AND (blood glucose OR blood lipids OR lipid metabolism) AND (randomized controlled trial OR controlled clinical trial RCT)	**2**
**CNKI**	(主题：FATmax) OR (主题：LIPOXmax) OR (主题：最大脂肪氧化强度) AND (主题：运动) OR (主题：训练) AND (主题：随机) OR (主题：对照) OR (主题：试验)	**3**

Note: ICTRP = International Clinical Trials Registry Platform; CNKI = Chinese National Knowledge Infrastructure.

SUPPLEMENTARY TABLE 4. Characteristics of the included studies

Study	Design	Participants (mean ± SD)	Health Status / Comorbidities	Training protocols							Outcome Measures
				%$\dot{V}O_{2max}$ at FATmax / HR at FATmax (bpm)	Training modes	Diet restriction	Warm-up	Single intervention duration (min)	Training frequency (days/week)	Intervention cycle (weeks)	
Cao et al. (2019) [15]	RCT	28W;<br>Age (years): 64.2 ± 5.1;<br>BMI (kg·m^{-2}): 27.1 ± 2.3;<br>$\dot{V}O_{2max}$ (mL/kg/min): 32.3 ± 5.4	Overweight	34.5 ± 8.0<br>101.0 ± 9.0	Running	N	Y	60	3	12	1. →TG<br>2. ↑HDL-C<br>3. →LDL-C<br>4. →TC
Jiang et al. (2020-a) [16]	CT	24W;<br>Age (years): 63.9 ± 6.1;<br>BMI (kg·m^{-2}): 26.6 ± 2.2;<br>$\dot{V}O_{2max}$ (mL/kg/min): 27.7 ± 4.1	Overweight + T2DM	41.3 ± 3.2<br>96.0 ± 12.0	Running	N	Y	60	3	16	1. →TG<br>2. ↑HDL-C<br>3. →LDL-C<br>4. →TC<br>5. ↓FPG<br>6. →HOMA-IR<br>7. →FINS
Jiang et al. (2020-b) [16]	CT	25M;<br>Age (years): 63.9 ± 6.1;<br>BMI (kg·m^{-2}): 26.9 ± 2.1;<br>$\dot{V}O_{2max}$ (mL/kg/min): 29.0 ± 5.2	Overweight + T2DM	46.1 ± 10.3<br>95.0 ± 12.0	Running	N	Y	60	3	16	1. →TG<br>2. ↑HDL-C<br>3. →LDL-C<br>4. →TC<br>5. ↓FPG<br>6. →HOMA-IR<br>7. →FINS
Jiang et al. (2020-c) [56]	RCT	29M;<br>Age (years): 59.1 ± 3.2;<br>BMI (kg·m^{-2}): 26.6 ± 2.6;<br>$\dot{V}O_{2max}$ (mL/kg/min): n/a	Overweight + MetS	43.5 ± 8.7<br>101.0 ± 9.2	RT	N	Y	60	3	12	1. ↓TG<br>2. ↑HDL-C<br>3. →LDL-C<br>4. ↓TC<br>5. ↓FPG<br>6. ↓HOMA-IR<br>7. ↓FINS
Ben Ounis et al. (2008-a) [50]	RCT	12W;<br>Age (years): 13.0 ± 0.4;<br>BMI (kg·m^{-2}): 30.0 ± 2.2;<br>$\dot{V}O_{2max}$ (mL/kg/min): n/a	Obese	n/a	Running	Y	Y	90	4	8	1. ↓TG<br>2. ↑HDL-C<br>3. ↓LDL-C<br>4. ↓TC<br>5. ↓FPG<br>6. →HOMA-IR<br>7. ↓FINS
Ben Ounis et al. (2008-b) [51]	RCT	16M;<br>Age (years): 13.3 ± 0.7;<br>BMI (kg·m^{-2}): 31.3 ± 4.0;<br>$\dot{V}O_{2max}$ (mL/kg/min): n/a	Obese	n/a<br>124.5 ± 3.6	Running	Y	Y	90	4	8	1. ↓TG<br>2. ↑HDL-C<br>3. ↓LDL-C<br>4. ↓TC<br>5. ↓FPG<br>6. ↓HOMA-IR<br>7. ↓FINS
Ben Ounis et al. (2009) [64]	RCT	18W;<br>Age (years): 13.1 ± 0.8;<br>BMI (kg·m^{-2}): 31.2 ± 2.1;<br>$\dot{V}O_{2max}$ (mL/kg/min): n/a	Obese	n/a	Cycling	Y	N	90	4	8	1. ↓FPG<br>2. ↓HOMA-IR<br>3. ↓FINS
Ben Ounis et al. (2010) [67]	RCT	32 (MW);<br>Age (years): 13.3 ± 0.4;<br>BMI (kg·m^{-2}): 31.3 ± 0.6;<br>$\dot{V}O_{2max}$ (mL/kg/min): n/a	Obese	66.0<br>136.1	Cycling	N	Y	90	4	8	1. ↓TG<br>2. ↑HDL-C<br>3. ↓FPG

SUPPLEMENTARY TABLE 4. Continue.

Study	Design	Participants (mean ± SD)	Health Status / Comorbidities	Training protocols: $\%\dot{V}O_{2max}$ at FATmax / HR at FATmax (bpm)	Training modes	Diet restriction	Warm-up	Single intervention duration (min)	Training frequency (days/week)	Intervention cycle (weeks)	Outcome Measures
Tan et al. (2012) [52]	RCT	48W; Age (years): 20–23; BMI (kg·m^{-2}): 27.5 ± 1.9; $\dot{V}O_{2max}$ (mL/kg/min): 34.1 ± 2.6	Overweight	54.0 ± 4.0<br>134.0 ± 3.0	Running	N	Y	60	5	8	1. ↓TG<br>2. ↓TC
Tan et al. (2015) [57]	RCT	37W; Age (years): 46–59; BMI (kg·m^{-2}): 28.2 ± 2.0; $\dot{V}O_{2max}$ (mL/kg/min): 26.0 ± 5.0	Overweight + MetS	42.2 ± 6.0<br>115.0 ± 6.2	Running	N	Y	50	3	24	1. ↓TG<br>2. →HDL-C<br>3. ↓LDL-C<br>4. →TC
Tan et al. (2016) [53]	RCT	26W; Age (years): 50.7 ± 5.5; BMI (kg·m^{-2}): 28.5 ± 2.1; $\dot{V}O_{2max}$ (mL/kg/min): 38.5 ± 7.5	Overweight	52.0 ± 6.0<br>106.0 ± 8.0	Running	N	Y	60	5	10	1. ↓TG<br>2. ↑HDL-C<br>3. ↓TC<br>4. ↓FPG
Tan et al. (2018) [54]	RCT	31W; Age (years): 63.0 ± 2.3; BMI (kg·m^{-2}): 26.6 ± 3.1; $\dot{V}O_{2max}$ (mL/kg/min): 38.5 ± 7.5	Overweight + T2DM	37.3 ± 7.3<br>100.0 ± 8.0	Running	N	Y	60	3	12	1. ↓TG<br>2. ↑HDL-C<br>3. ↓LDL-C<br>4. ↓TC<br>5. ↓FPG<br>6. ↓HOMA-IR<br>7. ↓FINS
Venables et al. (2008) [58]	CT	8M; Age (years): 39.0 ± 7.0; BMI (kg·m^{-2}): 32.5 ± 2.6; $\dot{V}O_{2max}$ (mL/kg/min): 33.4 ± 4.7	Obese	44.0 ± 2.0<br>n/a	RT	N	N	60	5	4	1. →FPG<br>2. →FINS
Lanzi et al. (2015) [59]	RCT	19M; Age (years): 38.1 ± 2.3; BMI (kg·m^{-2}): 40.9 ± 1.1; $\dot{V}O_{2max}$ (mL/kg/min): 23.1 ± 1.2	Obese	48.8 ± 2.9<br>≈ 123	Cycling	N	N	40–50	4	2	1. ↓FPG<br>2. ↓HOMA-IR<br>3. ↓FINS
Huang et al. (2018) [55]	RCT	32M; Age (years): 20.7 ± 1.1; BMI (kg·m^{-2}): 29.3 ± 3.2; $\dot{V}O_{2max}$ (mL/kg/min): 32.3 ± 4.6	Overweight	51.5 ± 7.3<br>n/a	Cycling	N	Y	40–60	3–5	12	1. →TG<br>2. ↑HDL-C<br>3. →LDL-C<br>4. →TC
Lu. (2023) [19]	RCT	25M; Age (years): 62.8 ± 3.6; BMI (kg·m^{-2}): 25.4 ± 2.6; $\dot{V}O_{2max}$ (mL/kg/min): 32.3 ± 3.1	Overweight	42.0 ± 6.3<br>106.3 ± 13.1	RT	N	Y	70	4	16	1. ↓TG<br>2. ↑HDL-C<br>3. ↓LDL-C<br>4. ↓TC<br>5. →FPG
Kantorowicz et al. (2021) [60]	CT	14W; Age (years): 30.1 ± 3.6; BMI (kg·m^{-2}): 33.9 ± 5.5; $\dot{V}O_{2max}$ (mL/kg/min): n/a	Obese + MetS	42.3 ± 8.7<br>114.2 ± 14.1	Running	N	Y	60	3	8	1. ↓FPG<br>2. ↓HOMA-IR<br>3. →FINS
Mohebbi et al. (2015) [61]	CT	9M; Age (years): 23.1 ± 1.1; BMI (kg·m^{-2}): 27.8 ± 1.4; $\dot{V}O_{2max}$ (mL/kg/min): 38.2 ± 3.4	Overweight	44.0 ± 6<br>135.6 ± 4.6	Cycling	N	Y	144	1	1	1. ↓FPG<br>2. ↓HOMA-IR<br>3. ↓FINS

SUPPLEMENTARY TABLE 4. Continue.

Study	Design	Participants (mean ± SD)	Health Status / Comorbidities	Training protocols							Outcome Measures
				$\%\dot{V}O_{2max}$ at FATmax / HR at FATmax (bpm)	Training modes	Diet restriction	Warm-up	Single intervention duration (min)	Training frequency (days/week)	Intervention cycle (weeks)	
Besnier et al. (2015) [62]	RCT	68W;<br>Age (years): 30.5 ± 5.9;<br>BMI (kg·m^{-2}): 33.3 ± 3.8;<br>$\dot{V}O_{2max}$ (mL/kg/min): 42.0 ± 6.7	Obese	45.7 ± 8.3<br>120.9 ± 13.6	Cycling	N	N	55	4	20	1. →TG<br>2. ↑HDL-C<br>3. ↓LDL-C<br>4. →TC<br>5. →FPG<br>6. ↓HOMA-IR<br>7. ↓FINS
Safarimosavi et al. (2021) [63]	RCT	16M;<br>Age (years): 39.1 ± 4.0;<br>BMI (kg·m^{-2}): 26.7 ± 3.0;<br>$\dot{V}O_{2max}$ (mL/kg/min): 30.5	Overweight + T2DM	41.0 ± 4.5<br>n/a	Cycling	N	Y	55	4	12	1. ↓FPG<br>2. ↓HOMA-IR<br>3. ↓FINS
Dumortier et al. (2003) [65]	CT	39 (MW);<br>Age (years): 52.0 ± 2.4;<br>BMI (kg·m^{-2}): 32.0 ± 1.7;<br>$\dot{V}O_{2max}$ (mL/kg/min): n/a	Obese + MetS	44.8 ± 3.7<br>n/a	Cycling	N	Y	40	3	8	1. →TG<br>2. →HDL-C<br>3. →LDL-C<br>4. →TC<br>5. →FPG<br>6. →FINS
Maurie et al. (2011) [66]	CT	51 (MW);<br>Age (years): 52.3 ± 2.0;<br>BMI (kg·m^{-2}): 32.1 ± 0.9;<br>$\dot{V}O_{2max}$ (mL/kg/min): 17.2 ± 0.9	Obese + T2DM	45.1 ± 2.9<br>n/a	RT	N	N	45	7	12	1. →TG<br>2. →HDL-C<br>3. →LDL-C<br>4. →TC<br>5. ↓FPG
Wang et al. (2023) [68]	CT	17W;<br>Age (years): 22.2 ± 3.4;<br>BMI (kg·m^{-2}): 26.1 ± 3.3;<br>$\dot{V}O_{2max}$ (mL/kg/min): 35.2 ± 7.6	Overweight	≈ 49<br>132.0 ± 18.6	Running	N	Y	45	4	8	1. →TG<br>2. →HDL-C<br>3. →LDL-C<br>4. →TC
Elloumi et al. (2009) [69]	RCT	14M;<br>Age (years): 13.2 ± 0.9;<br>BMI (kg·m^{-2}): 31.6 ± 3.3;<br>$\dot{V}O_{2max}$ (mL/kg/min): n/a	Obese	n/a	Running	Y	Y	90	4	8	1. ↓HOMA-IR<br>2. ↓FINS

SUPPLEMENTARY TABLE 5. Methodological quality assessment [PEDro]

Author, year	D1	D2	D3	D4	D5	D6	D7	D8	D9	D10	D11	Total
Cao et al. (2019)	Y	1	0	1	0	0	0	1	0	1	1	5
Jiang et al. (2020-a)	Y	1	0	1	0	0	0	1	0	1	1	5
Jiang et al. (2020-b)	Y	1	0	1	0	0	0	1	0	1	1	5
Jiang et al. (2020-c)	Y	1	0	1	0	1	0	1	1	1	1	7
Ben Ounis et al. (2008-a)	Y	1	0	1	0	0	0	1	1	1	1	6
Ben Ounis et al. (2008-b)	Y	1	0	1	0	0	0	1	1	1	1	6
Ben Ounis et al. (2009)	Y	1	0	1	0	0	0	1	1	1	1	6
Ben Ounis et al. (2010)	Y	1	0	1	0	0	0	1	1	1	1	6
Tan et al. (2012)	Y	1	0	1	0	0	0	1	0	1	1	5
Tan et al. (2015)	Y	1	0	1	0	1	0	1	0	1	1	6
Tan et al. (2016)	Y	1	0	1	0	0	0	1	0	1	1	5
Tan et al. (2018)	Y	1	0	1	0	1	0	1	1	1	1	7
Venables et al. (2008)	Y	1	0	1	0	1	0	1	0	1	1	6
Lanzi et al. (2015)	Y	1	1	1	0	1	0	1	0	1	1	7
Huang et al. (2018)	Y	1	0	1	0	1	0	1	0	1	1	6
Lu. (2023)	Y	1	0	1	0	0	0	1	0	1	1	5
Kantorowicz et al. (2021)	Y	1	0	1	0	1	0	1	0	1	1	6
Mohebbi et al. (2015)	Y	1	0	1	0	1	0	1	0	1	1	6
Besnier et al. (2015)	Y	1	0	1	0	0	0	1	1	1	1	6
Safarimosa et al. (2021)	Y	1	0	1	0	1	0	1	0	1	1	6
Dumortier et al. (2003)	Y	1	1	1	0	1	0	1	0	1	1	7
Maurie et al. (2011)	Y	1	0	1	0	1	0	1	0	1	1	6
Wang et al. (2023)	Y	1	0	1	0	1	0	1	0	1	1	6
Elloumi et al. (2009)	Y	1	0	1	0	0	0	1	1	1	1	6

Note: Studies scoring ≥ 6 were considered high quality, those scoring 4–5 moderate quality, and those scoring ≤ 3 low quality. The PEDro scale includes 11 items: (1) eligibility criteria were specified (not included in total score); (2) subjects were randomly allocated; (3) allocation was concealed; (4) groups were similar at baseline; (5) blinding of subjects; (6) blinding of therapists; (7) blinding of assessors; (8) ≥ 85% follow-up; (9) intention-to-treat analysis; (10) between-group comparisons reported; and (11) both point estimates and variability measures provided.

SUPPLEMENTARY TABLE 6. SD_{diff} vs. pooled SD of baseline scores

Outcome	SDdiff				pooled SD of baseline scores			
	Hedges' *g* (95%CI)	*p*-value	I^2	PI	Hedges' *g* (95%CI)	*p*-value	I^2	PI
TG	-0.55 (-0.83, -0.27)	< 0.01	48%	(-1.40, 0.30)	-0.56 (-0.85, -0.27)	< 0.01	58%	(-1.49, 0.37)
HDL-C	0.51 (0.02, 0.99)	0.03	82%	(-1.28, 2.29)	0.53 (0.04, 1.02)	0.03	84%	(-1.31, 2.36)
LDL-C	-0.18 (-0.50, 0.14)	0.28	49%	(-1.12, 0.76)	-0.17 (-0.57, 0.23)	0.40	73%	(-1.48, 1.14)
TC	-0.23 (-0.42, -0.03)	0.02	3%	(-0.47, 0.02)	-0.22 (-0.41, -0.03)	0.02	8%	(-0.51, 0.07)
FPG	-1.05 (-1.46, -0.64)	< 0.01	74%	(-2.60, 0.49)	-1.15 (-1.60, -0.70)	< 0.01	80%	(-2.92, 0.62)
HOMA-IR	-0.82 (-1.17, -0.48)	< 0.01	45%	(-1.71, 0.06)	-0.86 (-1.22, -0.50)	< 0.01	52%	(-1.84, 0.11)
FINS	-0.75 (-1.07, -0.44)	< 0.01	45%	(-1.61, 0.10)	-0.80 (-1.14, -0.46)	< 0.01	54%	(-1.80, 0.20)

Note: SD_{diff} refer SD difference scores corrected for correlation, used correlated groups, report for use in meta-analyses. An formula to calculate the SD_{diff} from the standard deviations of both groups and their correlation as:

$$SD_{diff} = \sqrt{SD_{pre}^2 + SD_{post}^2 - 2r \times SD_{pre} \times SD_{post}}$$

where SD_{diff} is the standard deviation of the difference in means, SD_{pre} is the standard deviation from pre-intervention, and SD_{post} is the standard deviation from post-intervention. The pooled SD of Baseline Scores refer $(SD_{pre} + SD_{post})/2$, used correlated groups, report for use in meta-analyses.

TG Study	FATmax N	FATmax Mean	FATmax SD	CON N	CON Mean	CON SD	SMD [95% CI]
Cao et al.(2019)	13	-0.45	1.46	15	0.09	1.31	-0.38 [-1.16, 0.40]
Jiang et al.(2020-a)	11	-0.55	1.47	13	-0.14	1.06	-0.31 [-1.17, 0.55]
Jiang et al.(2020-b)	14	-0.48	1.13	11	-0.12	1.25	-0.29 [-1.13, 0.54]
Jiang et al.(2020-c)	15	-0.40	0.78	14	-0.04	0.64	-0.49 [-1.25, 0.27]
Ben Ounis et al.(2008-a)	6	-0.28	0.17	6	-0.06	0.12	-1.36 [-2.75, 0.03]
Ben Ounis et al.(2008-b)	8	-0.24	0.20	8	-0.02	0.17	-1.12 [-2.24, 0.01]
Ben Ounis et al.(2010)	16	-0.29	0.11	16	-0.02	0.11	-2.34 [-3.27, -1.41]
Tan et al.(2012)	29	-0.48	0.70	19	0.01	0.51	-0.79 [-1.39, -0.20]
Tan et al.(2015)	18	-0.29	0.43	19	0.01	0.53	-0.61 [-1.28, 0.07]
Tan et al.(2016)	15	-0.32	0.38	11	0.01	0.48	-0.73 [-1.59, 0.12]
Tan et al.(2018)	16	-0.51	0.65	15	0.02	0.46	-0.92 [-1.69, -0.16]
Huang et al.(2018)	16	0.07	0.53	16	0.26	0.47	-0.37 [-1.09, 0.35]
Lu.(2023)	12	-0.42	0.75	13	0.03	0.84	-0.55 [-1.38, 0.28]
Besnier et al.(2015)	33	-0.10	0.36	35	-0.20	0.53	0.22 [-0.26, 0.70]
Dumortier et al.(2003)	28	-0.01	0.24	11	-0.13	0.46	0.32 [-0.50, 1.15]
Maurie et al.(2011)	39	-0.07	0.20	12	-0.02	0.42	-0.15 [-0.93, 0.63]
Wang et al.(2023)	9	-0.17	0.60	8	0.20	0.58	-0.60 [-1.63, 0.44]
**RE Model**							-0.55 [-0.83, -0.27]

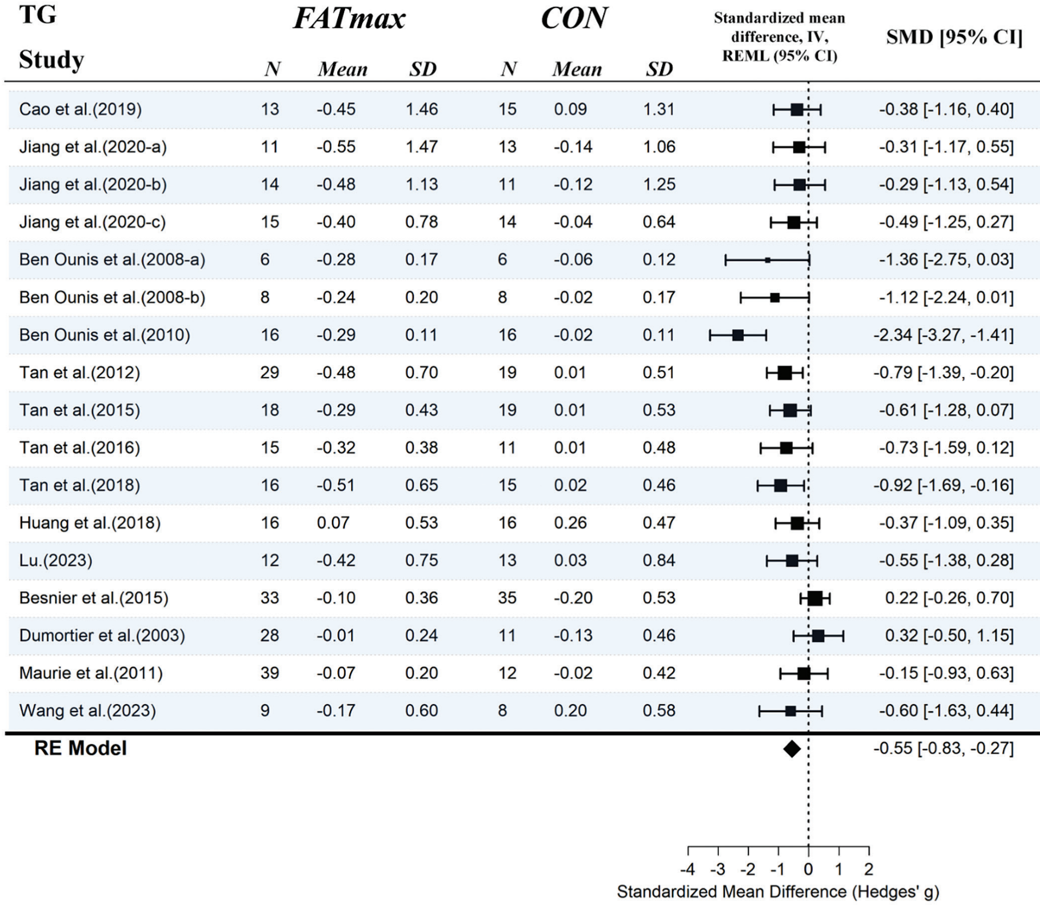

HDL-C Study	FATmax N	FATmax Mean	FATmax SD	CON N	CON Mean	CON SD	SMD [95% CI]
Cao et al.(2019)	13	0.13	0.18	15	-0.05	0.25	0.81 [0.01, 1.60]
Jiang et al.(2020-a)	11	0.33	0.33	13	-0.02	0.36	0.98 [0.10, 1.87]
Jiang et al.(2020-b)	14	0.38	0.44	11	0.03	0.41	0.80 [-0.05, 1.65]
Jiang et al.(2020-c)	15	0.27	0.27	14	0.01	0.32	0.85 [0.06, 1.65]
Ben Ounis et al.(2008-a)	6	0.15	0.14	6	-0.02	0.12	1.22 [-0.13, 2.57]
Ben Ounis et al.(2008-b)	8	0.15	0.12	8	-0.03	0.10	1.54 [0.34, 2.74]
Ben Ounis et al.(2010)	16	0.17	0.06	16	-0.03	0.08	2.81 [1.80, 3.83]
Tan et al.(2015)	18	0.06	0.27	19	0.01	0.29	0.18 [-0.49, 0.84]
Tan et al.(2016)	15	0.04	0.29	11	0.01	0.21	0.12 [-0.67, 0.91]
Tan et al.(2018)	16	0.06	0.21	15	0.00	0.21	0.28 [-0.45, 1.01]
Huang et al.(2018)	16	0.22	0.19	16	0.11	0.20	0.55 [-0.18, 1.28]
Lu.(2023)	12	0.31	0.24	13	0.01	0.25	1.19 [0.30, 2.07]
Besnier et al.(2015)	33	0.10	0.30	35	0.10	0.30	0.00 [-0.48, 0.48]
Dumortier et al.(2003)	28	0.03	0.13	11	0.21	0.09	-1.60 [-2.35, -0.84]
Maurie et al.(2011)	39	-0.05	0.13	12	0.10	0.20	-0.89 [-1.67, -0.10]
Wang et al.(2023)	9	0.03	0.32	8	0.03	0.23	0.00 [-1.00, 1.00]
**RE Model**							0.51 [0.02, 0.99]

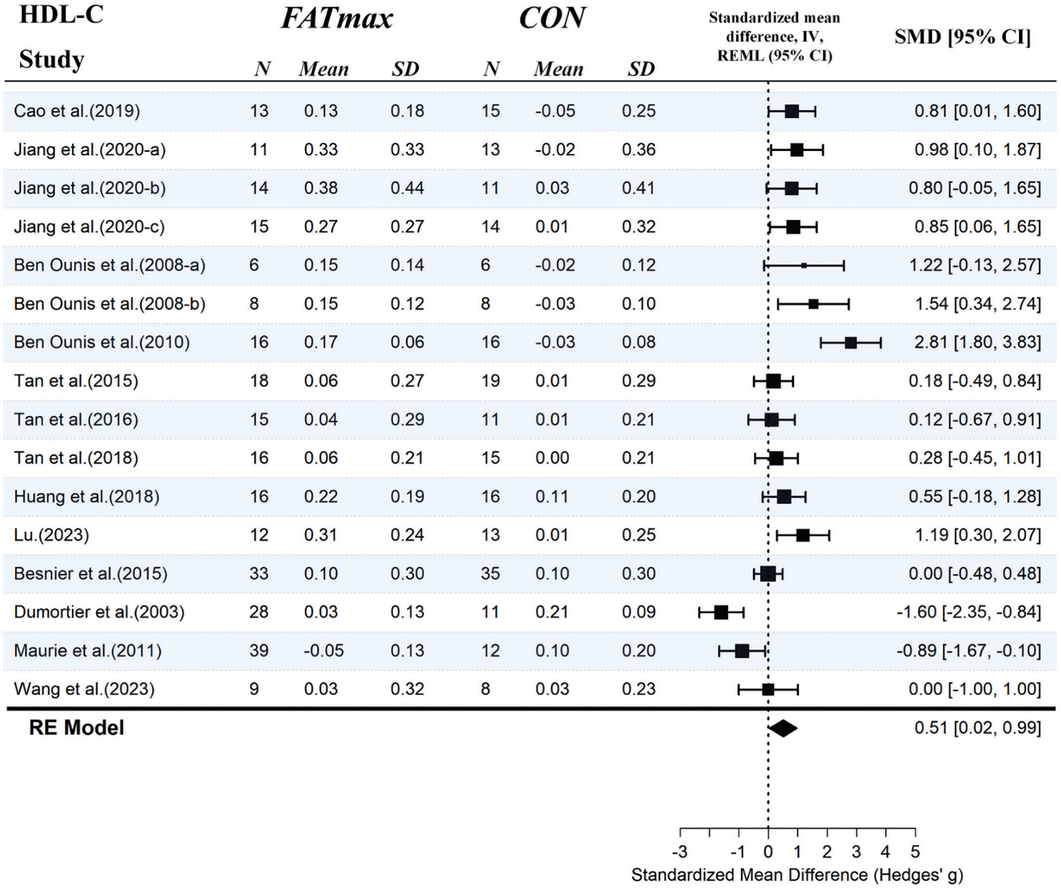

SUPPLEMENTARY FIG. 2. (Forest plots)

LDL-C Study	FATmax N	FATmax Mean	FATmax SD	CON N	CON Mean	CON SD	SMD [95% CI]
Cao et al.(2019)	13	-0.04	0.72	15	0.11	0.83	-0.19 [-0.96, 0.58]
Jiang et al.(2020-a)	11	-0.06	0.74	13	0.38	0.68	-0.60 [-1.46, 0.27]
Jiang et al.(2020-b)	14	-0.20	0.77	11	-0.07	0.62	-0.18 [-0.99, 0.64]
Jiang et al.(2020-c)	15	-0.39	0.63	14	-0.08	0.79	-0.42 [-1.19, 0.34]
Ben Ounis et al.(2008-a)	6	-0.68	0.23	6	-0.38	0.22	-1.24 [-2.60, 0.11]
Ben Ounis et al.(2008-b)	8	-0.55	0.18	8	-0.27	0.23	-1.26 [-2.41, -0.11]
Tan et al.(2015)	18	-0.19	0.64	19	-0.01	0.88	-0.23 [-0.89, 0.43]
Tan et al.(2018)	16	-0.20	0.73	15	0.05	0.67	-0.35 [-1.08, 0.39]
Huang et al.(2018)	16	-0.19	0.65	16	-0.06	0.51	-0.22 [-0.94, 0.50]
Lu.(2023)	12	-0.45	0.69	13	0.01	0.62	-0.68 [-1.52, 0.16]
Besnier et al.(2015)	33	-0.10	0.70	35	-0.30	0.75	0.27 [-0.21, 0.76]
Dumortier et al.(2003)	28	0.18	0.22	11	-0.36	0.36	1.77 [0.79, 2.76]
Maurie et al.(2011)	39	0.08	0.22	12	-0.03	0.32	0.39 [-0.35, 1.14]
Wang et al.(2023)	9	-0.09	0.41	8	0.08	0.60	-0.32 [-1.35, 0.72]
**RE Model**							-0.18 [-0.50, 0.14]

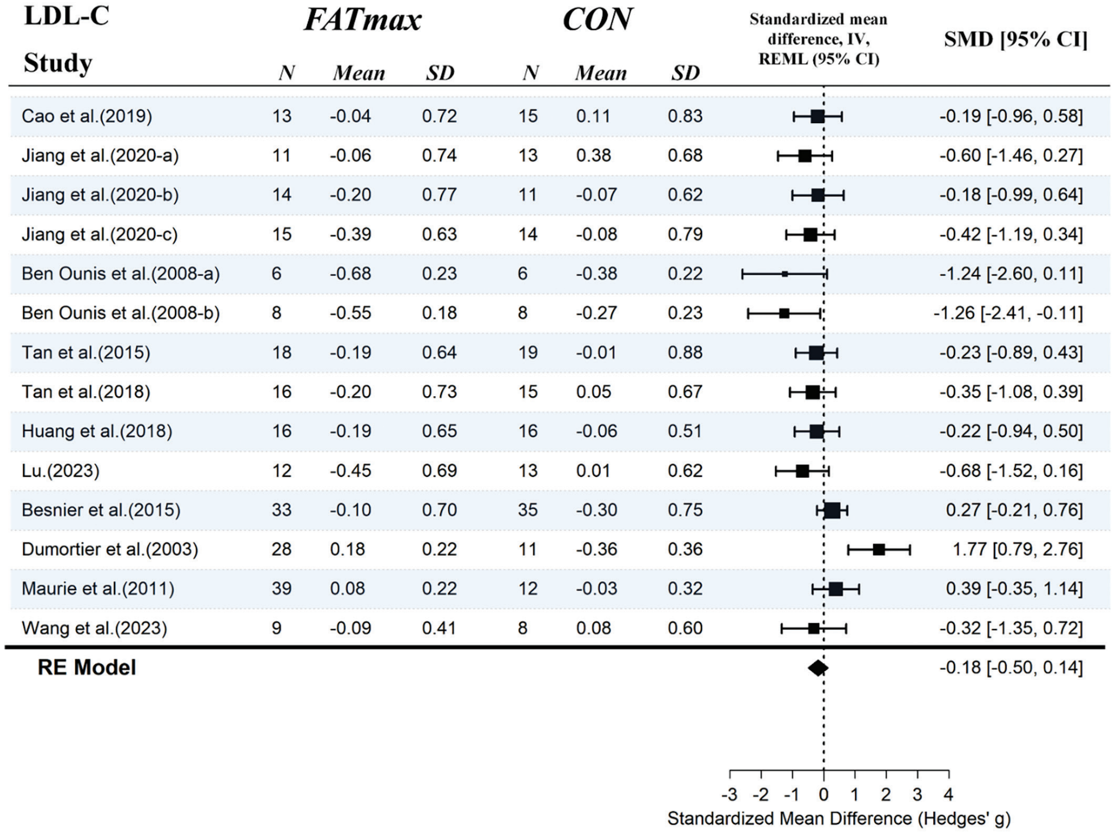

TC Study	FATmax N	FATmax Mean	FATmax SD	CON N	CON Mean	CON SD	SMD [95% CI]
Cao et al.(2019)	13	-0.04	1.03	15	0.11	0.75	-0.16 [-0.94, 0.62]
Jiang et al.(2020-a)	11	0.20	0.83	13	0.19	1.00	0.01 [-0.82, 0.84]
Jiang et al.(2020-b)	14	0.01	0.96	11	0.12	1.04	-0.11 [-0.94, 0.72]
Jiang et al.(2020-c)	15	-0.40	0.80	14	0.00	0.79	-0.49 [-1.25, 0.28]
Ben Ounis et al.(2008-a)	6	-0.66	0.53	6	-0.43	0.50	-0.41 [-1.67, 0.84]
Ben Ounis et al.(2008-b)	8	-0.51	0.55	8	-0.31	0.55	-0.34 [-1.40, 0.71]
Tan et al.(2012)	29	-0.45	0.54	19	0.09	0.56	-0.96 [-1.59, -0.33]
Tan et al.(2015)	18	-0.18	0.49	19	-0.01	0.77	-0.26 [-0.92, 0.40]
Tan et al.(2016)	15	-0.11	0.50	11	-0.02	0.62	-0.15 [-0.98, 0.67]
Tan et al.(2018)	16	-0.35	0.68	15	0.05	0.57	-0.62 [-1.37, 0.12]
Huang et al.(2018)	16	0.08	0.73	16	0.20	0.53	-0.18 [-0.90, 0.53]
Lu.(2023)	12	-0.08	0.62	13	-0.06	0.68	-0.03 [-0.85, 0.79]
Besnier et al.(2015)	33	-0.10	0.85	35	-0.20	0.85	0.12 [-0.37, 0.60]
Dumortier et al.(2003)	28	0.10	0.25	11	0.05	0.57	0.11 [-0.72, 0.94]
Maurie et al.(2011)	39	-0.11	0.26	12	-0.13	0.36	0.06 [-0.66, 0.79]
Wang et al.(2023)	9	-0.25	0.51	8	0.06	0.71	-0.48 [-1.52, 0.57]
**RE Model**							-0.23 [-0.42, -0.03]

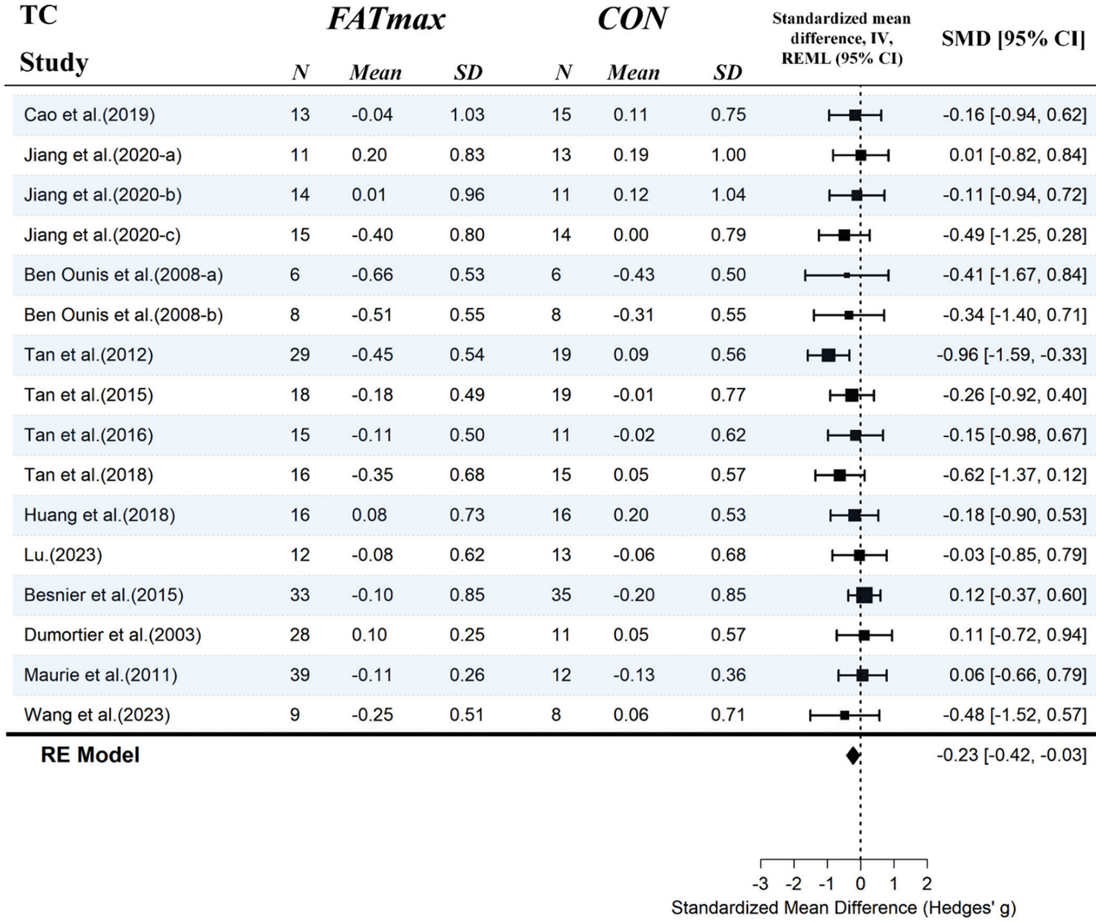

SUPPLEMENTARY FIG. 3. (Forest plots)

FPG Study	FATmax N	FATmax Mean	FATmax SD	CON N	CON Mean	CON SD	Standardized mean difference, IV, REML (95% CI)	SMD [95% CI]
Jiang et al.(2020-a)	13	-0.59	1.47	15	0.22	1.34		-0.56 [-1.35, 0.23]
Jiang et al.(2020-b)	14	-0.87	1.10	11	0.43	1.19		-1.10 [-1.99, -0.21]
Jiang et al.(2020-c)	15	-1.08	1.31	14	-0.24	1.54		-0.57 [-1.34, 0.20]
Ben Ounis et al.(2008-a)	6	-0.36	0.15	6	-0.06	0.15		-1.84 [-3.32, -0.36]
Ben Ounis et al.(2008-b)	8	-0.32	0.19	8	-0.08	0.16		-1.27 [-2.42, -0.12]
Ben Ounis et al.(2009)	9	-0.36	0.14	9	-0.05	0.13		-2.23 [-3.47, -0.98]
Ben Ounis et al.(2010)	16	-0.58	0.60	16	-0.04	0.51		-0.95 [-1.71, -0.20]
Tan et al.(2016)	15	-0.75	0.33	11	-0.01	0.38		-2.03 [-3.04, -1.01]
Tan et al.(2018)	16	-0.96	1.15	15	0.06	1.10		-0.88 [-1.65, -0.12]
Venables et al.(2008)	8	-0.30	0.20	8	-0.10	0.20		-0.95 [-2.05, 0.16]
Lanzi et al.(2015)	10	-0.10	0.10	9	-0.20	0.20		0.60 [-0.40, 1.60]
Huang et al.(2018)	16	0.22	0.42	16	0.27	0.35		-0.13 [-0.84, 0.59]
Lu.(2023)	12	-0.36	0.70	13	-0.16	0.64		-0.29 [-1.11, 0.54]
Kantorowicz et al.(2021)	14	-0.06	0.57	14	0.06	0.82		-0.16 [-0.93, 0.61]
Mohebbi et al.(2015)	9	-0.50	0.10	9	-0.20	0.10		-2.86 [-4.25, -1.46]
Besnier et al.(2015)	33	-0.10	0.40	35	0.00	0.46		-0.23 [-0.71, 0.25]
Safarimosav et al.(2021)	8	-0.75	0.27	8	0.15	0.22		-3.42 [-5.09, -1.75]
Dumortier et al.(2003)	28	-0.69	0.65	11	0.79	0.85		-1.91 [-2.86, -0.96]
Maurie et al.(2011)	39	-0.61	0.28	12	0.22	0.62		-1.69 [-2.68, -0.71]
**RE Model**								-1.05 [-1.46, -0.64]

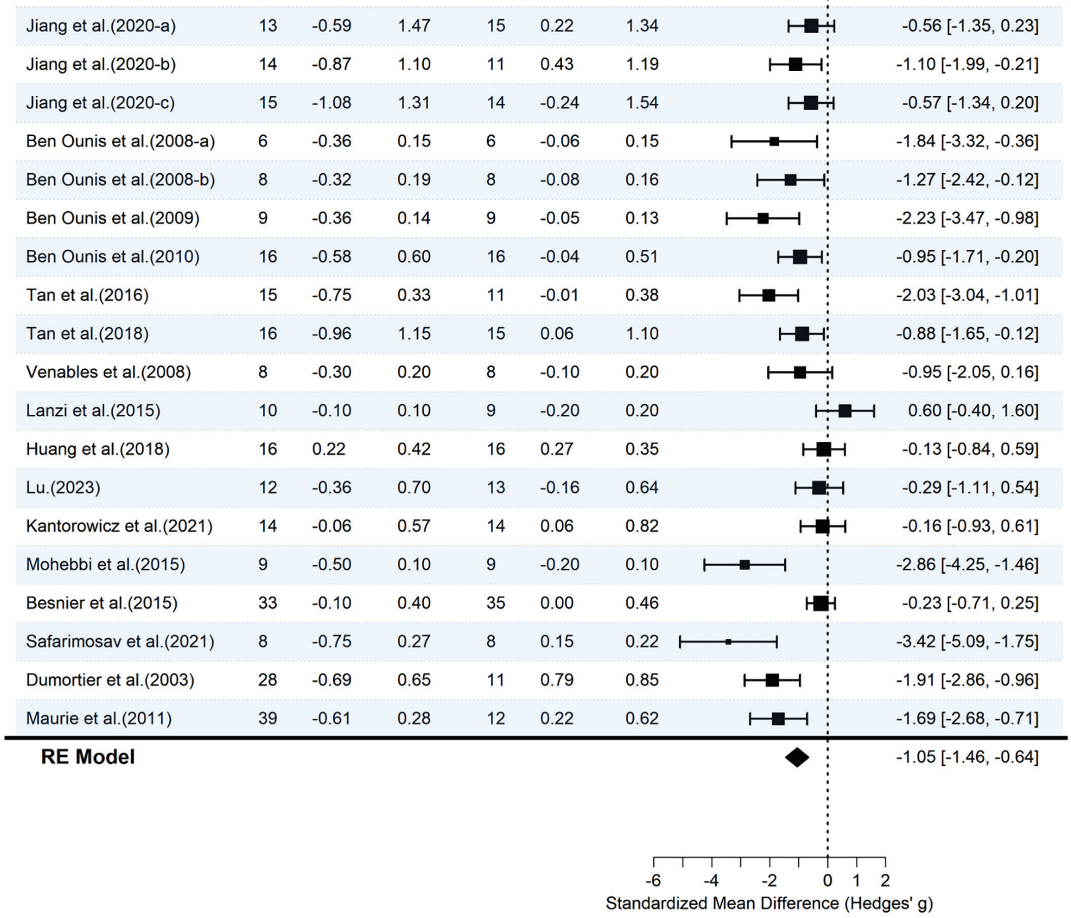

HOMA-IR Study	FATmax N	FATmax Mean	FATmax SD	CON N	CON Mean	CON SD	Standardized mean difference, IV, REML (95% CI)	SMD [95% CI]
Jiang et al.(2020-a)	11	-0.85	3.67	13	0.34	3.11		-0.34 [-1.19, 0.51]
Jiang et al.(2020-b)	14	-0.95	2.08	11	-0.10	3.23		-0.30 [-1.15, 0.55]
Jiang et al.(2020-c)	15	-1.09	1.19	14	-0.39	1.87		-0.43 [-1.21, 0.34]
Ben Ounis et al.(2008-a)	6	-2.01	1.51	6	-0.29	1.61		-1.02 [-2.33, 0.30]
Ben Ounis et al.(2008-b)	8	-2.13	1.37	8	-0.24	1.75		-1.13 [-2.27, -0.00]
Ben Ounis et al.(2009)	9	-1.95	1.31	9	-0.18	1.47		-1.21 [-2.28, -0.14]
Tan et al.(2018)	16	-1.78	1.97	15	0.06	2.16		-0.87 [-1.63, -0.10]
Lanzi et al.(2015)	10	-0.80	0.36	9	-0.10	0.40		-1.76 [-2.88, -0.63]
Kantorowicz et al.(2021)	14	0.10	1.35	14	-0.13	1.95		0.13 [-0.64, 0.90]
Mohebbi et al.(2015)	9	-0.40	0.20	9	-0.10	0.20		-1.43 [-2.53, -0.33]
Besnier et al.(2015)	33	-1.60	2.41	35	-0.40	1.55		-0.59 [-1.08, -0.09]
Safarimosav et al.(2021)	8	-1.48	0.53	8	0.27	0.53		-3.13 [-4.69, -1.56]
Elloumi et al.(2009)	7	-1.56	1.14	7	-0.52	1.15		-0.85 [-2.03, 0.33]
**RE Model**								-0.82 [-1.17, -0.48]

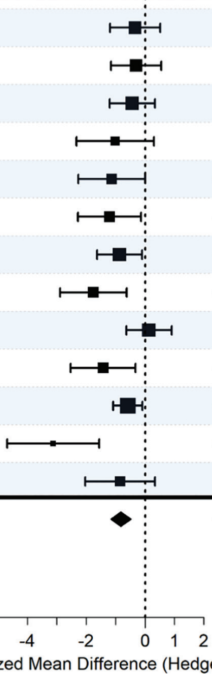

SUPPLEMENTARY FIG. 4. (Forest plots)

FINS Study	*FATmax* N	*FATmax* Mean	*FATmax* SD	*CON* N	*CON* Mean	*CON* SD	SMD [95% CI]
Jiang et al.(2020-a)	11	-0.70	8.85	13	0.45	7.33	-0.14 [-0.98, 0.71]
Jiang et al.(2020-b)	14	-1.32	6.26	11	-0.65	7.67	-0.09 [-0.93, 0.74]
Jiang et al.(2020-c)	15	-2.94	5.59	14	-0.39	4.91	-0.47 [-1.23, 0.29]
Ben Ounis et al.(2008-a)	6	-8.90	4.96	6	-1.20	5.03	-1.42 [-2.81, -0.04]
Ben Ounis et al.(2008-b)	8	-9.40	4.11	8	-0.80	5.50	-1.67 [-2.90, -0.44]
Ben Ounis et al.(2009)	9	-8.40	3.86	9	-0.70	4.41	-1.77 [-2.93, -0.61]
Tan et al.(2018)	16	-3.33	5.22	15	0.06	5.29	-0.63 [-1.37, 0.12]
Venables et al.(2008)	8	-1.00	5.15	8	-0.60	9.81	-0.05 [-1.10, 1.00]
Lanzi et al.(2015)	10	-6.60	2.82	9	-0.50	3.24	-1.92 [-3.08, -0.76]
Kantorowicz et al.(2021)	14	-0.22	6.10	14	0.42	7.30	-0.09 [-0.86, 0.68]
Mohebbi et al.(2015)	9	-0.70	0.92	9	-0.10	0.60	-0.74 [-1.76, 0.28]
Besnier et al.(2015)	33	-6.80	9.37	35	-1.90	6.81	-0.59 [-1.09, -0.09]
Safarimosav et al.(2021)	8	-3.90	1.75	8	0.70	1.37	-2.76 [-4.25, -1.28]
Dumortier et al.(2003)	28	-7.43	4.60	11	-5.10	2.22	-0.63 [-1.26, -0.00]
Elloumi et al.(2009)	7	-6.40	4.60	7	-1.76	5.15	-0.89 [-2.08, 0.30]
**RE Model**							-0.75 [-1.07, -0.44]

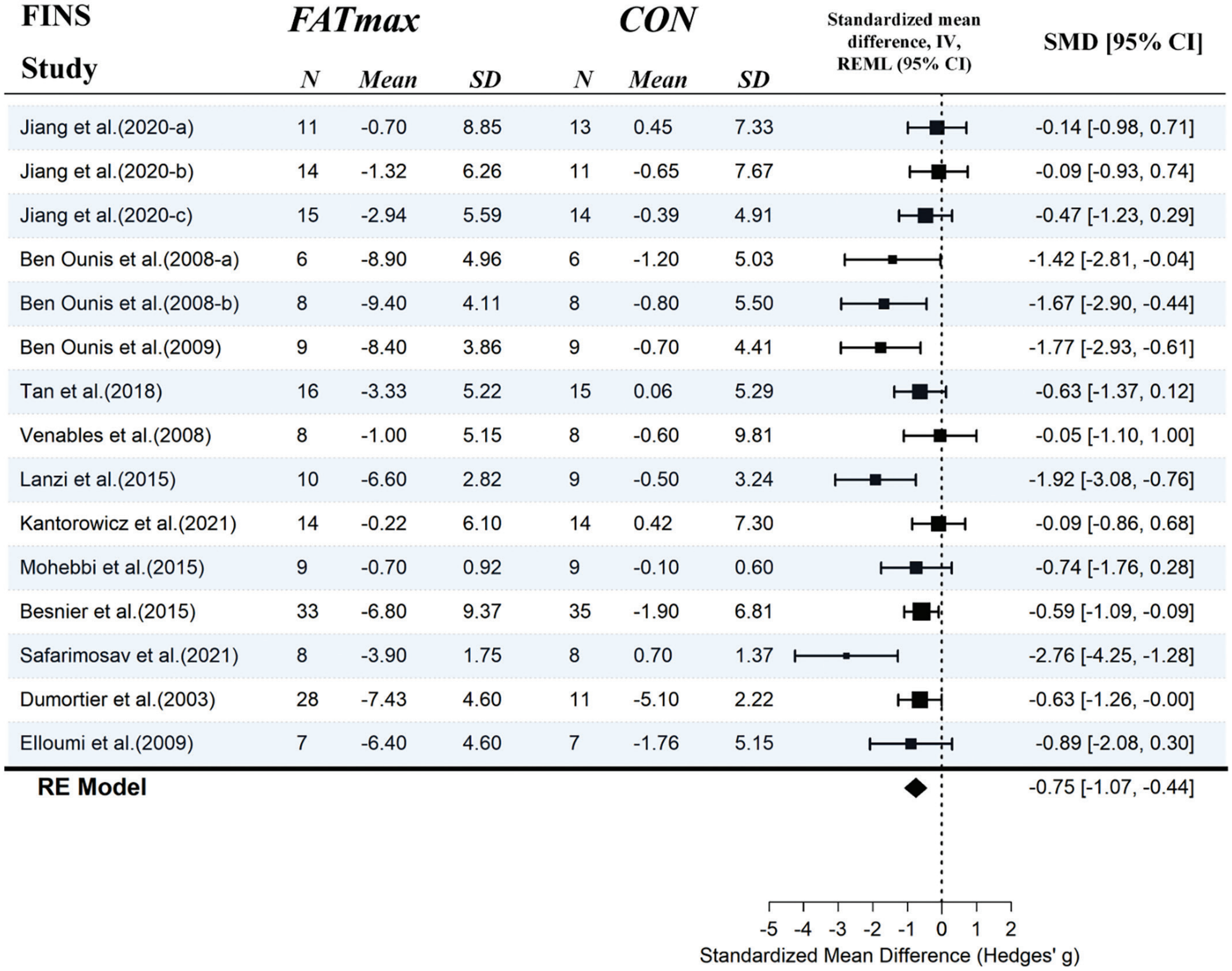

SUPPLEMENTARY FIG. 5. (Forest plots)

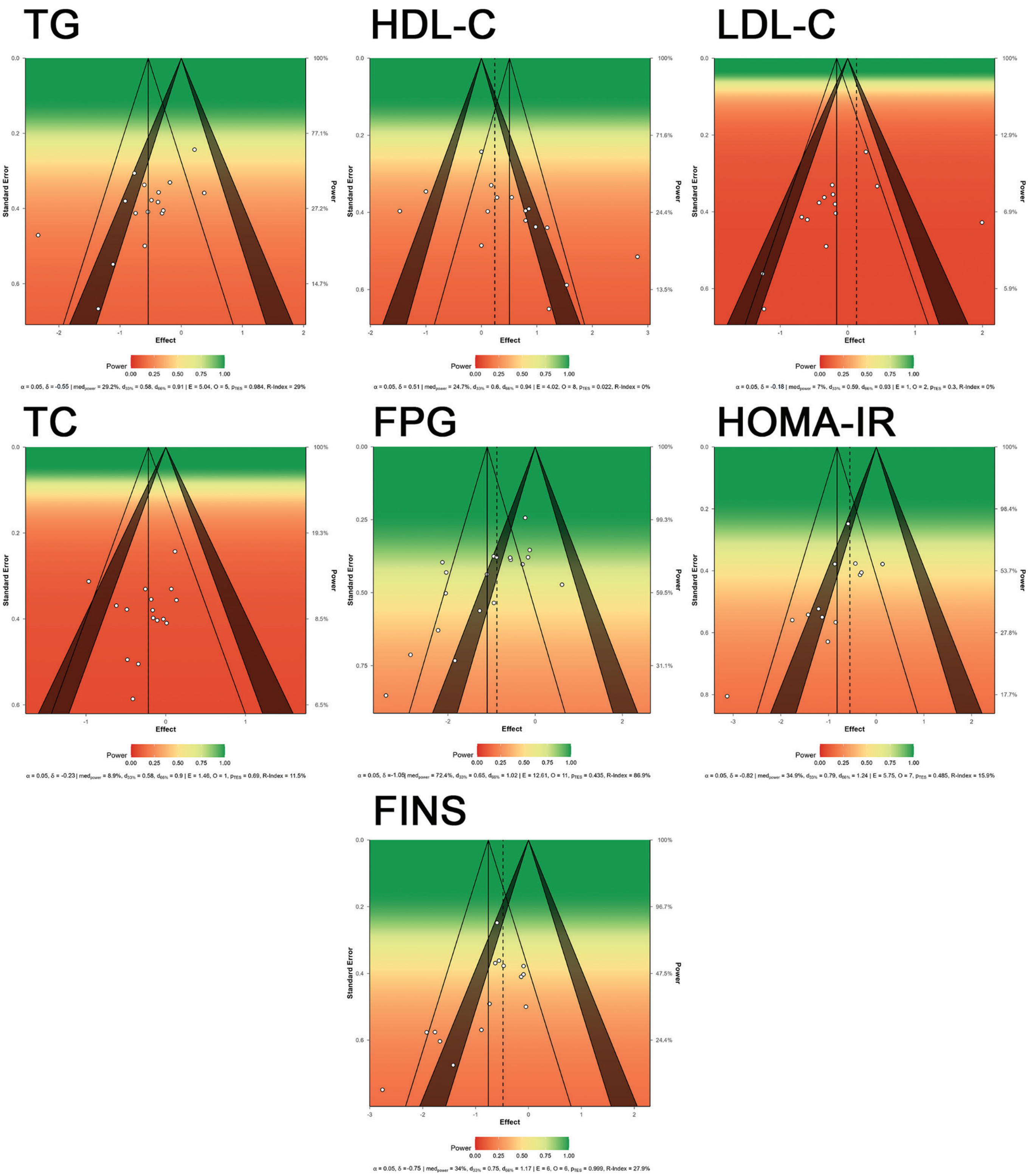

SUPPLEMENTARY FIG. 6. (Power visualization) The vertical solid line represents the pooled effect size, and the vertical dash line represents the adjusted pooled effect size. Significance contours at .05 and .01 levels are noted by the shaded area. Median power indicates the median power of all included effect sizes. $d_{33\%}$ and $d_{66\%}$ indicate the true effect sizes necessary for achieving 33% and 66% levels of median power. E, O, and PTES show the results of a test of excess significance. R-index denotes the expected replicability of findings.

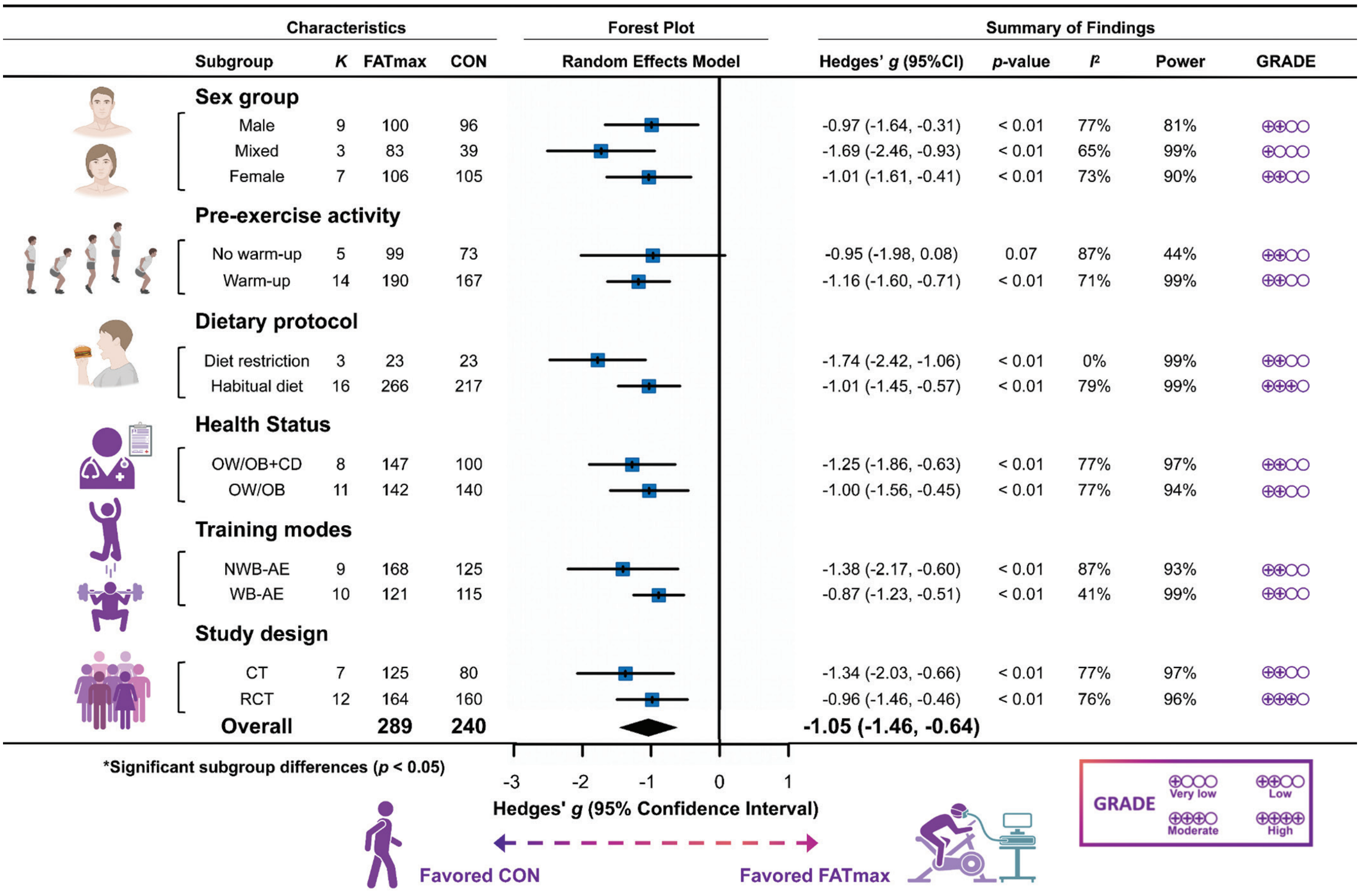

SUPPLEMENTARY FIG. 7. Subgroup analyses based on FPG

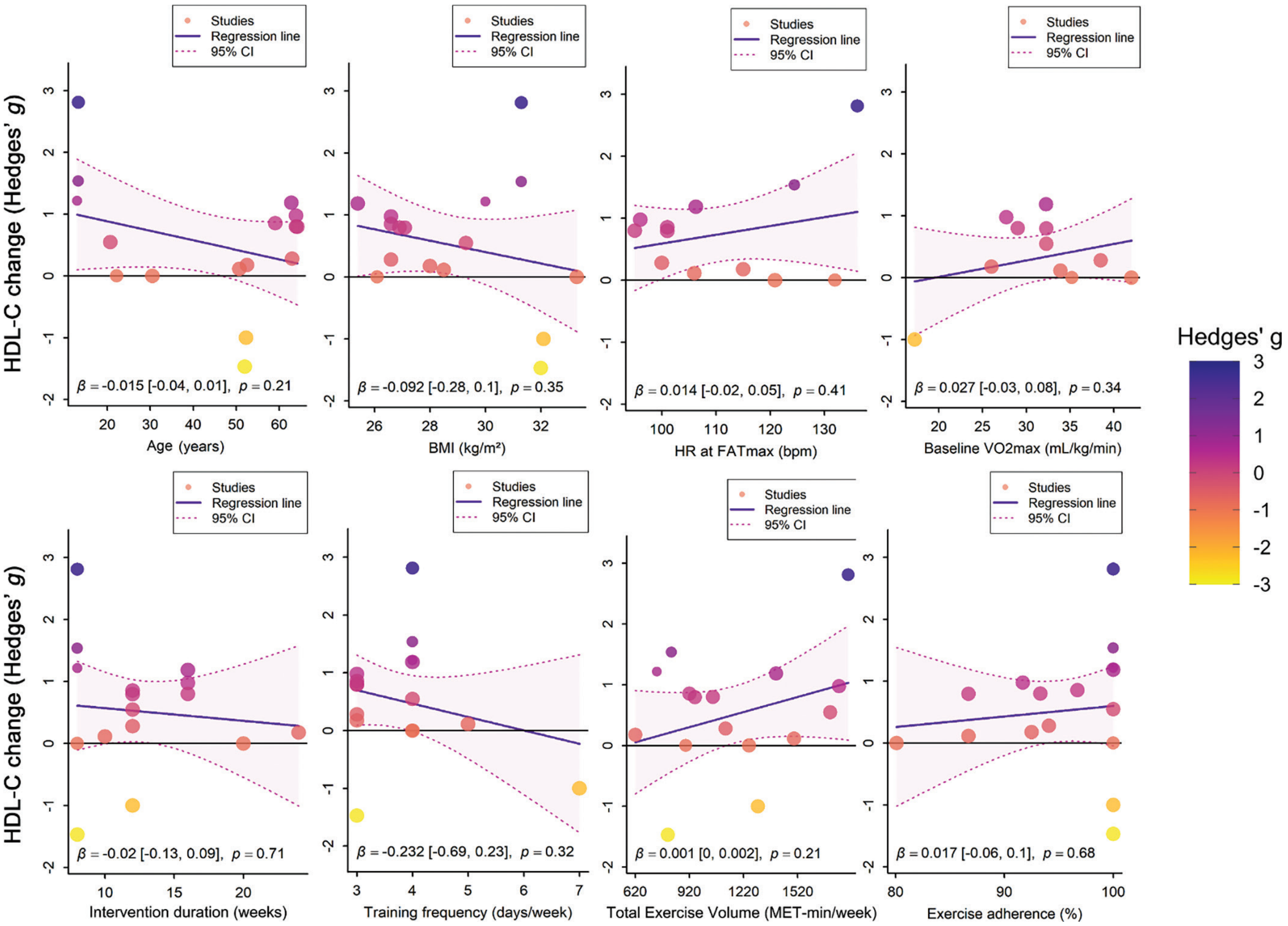

SUPPLEMENTARY FIG. 8. The bubble plots illustrate the relationship between FATmax-induced HDL-C changes (Hedges' *g*) and participant characteristics (Age, BMI, Baseline $\dot{V}O_{2max}$) or training parameters (HR at FATmax, Intervention duration, Training frequency, Total Exercise Volume, and Exercise adherence). Each bubble represents an individual study, colored according to the magnitude of the effect size (see color scale on the right). The vertical axis represents the change in HDL-C. The solid purple line indicates the linear regression slope, while the pink shaded area (bounded by dotted lines) represents the 95% confidence interval (CI). The regression coefficient (β), its 95% CI, and the p-value are reported within each panel.

Group		K	Hedges' g	95% CI	p-value	p-diff
Pattern 1	Low HDL-C × Short session duration	2	0.21	[-0.31, 0.73]	0.43	< 0.01
	High HDL-C × Short session duration	4	-0.58	[-1.37, 0.21]	0.15	
	Low HDL-C × Long session duration	7	1.19	[0.60, 1.78]	< 0.01	
	High HDL-C × Long session duration	3	0.61	[0.09, 1.13]	0.02	
Pattern 2	Low HDL-C × Low intensity	4	0.56	[0.11, 1.00]	0.01	< 0.01
	High HDL-C × Low intensity	1	n/a	n/a	n/a	
	Low HDL-C × High intensity	6	1.10	[0.30, 1.90]	< 0.01	
	High HDL-C × High intensity	5	-0.33	[-1.14, 0.48]	0.43	
Pattern 3	Short session duration × Low intensity	1	n/a	n/a	n/a	< 0.01
	Long session duration × Low intensity	4	0.76	[0.37, 1.15]	< 0.01	
	Short session duration × High intensity	5	-0.38	[-1.08, 0.32]	0.29	
	Long session duration × High intensity	6	1.18	[0.44, 1.93]	< 0.01	

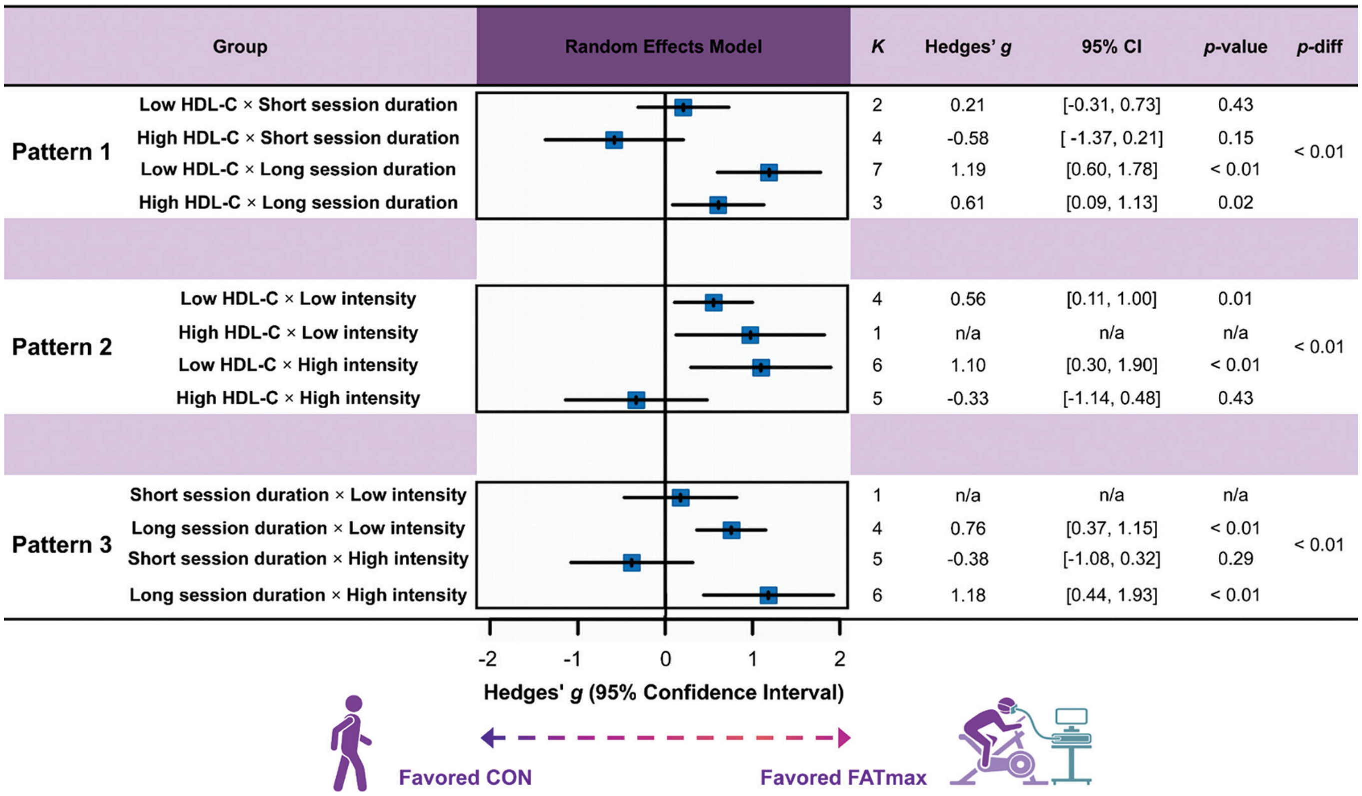

SUPPLEMENTARY FIG. 9. This figure illustrates the combined subgroup analyses for HDL-C. Subgroups were stratified based on specific thresholds derived from meta-regression analysis: (1) Baseline HDL-C levels were classified as Low (≤ 1.36 mmol/L) or High (> 1.36 mmol/L); (2) Exercise intensity was categorized as Low (< 42.2% $\dot{V}O_{2max}$) or High (≥ 42.2% $\dot{V}O_{2max}$); and (3) Session duration was defined as Short (< 60 min) or Long (≥ 60 min). *K* denotes the number of studies included in each subgroup; *p*-diff indicates the *p*-value for the difference between subgroups.

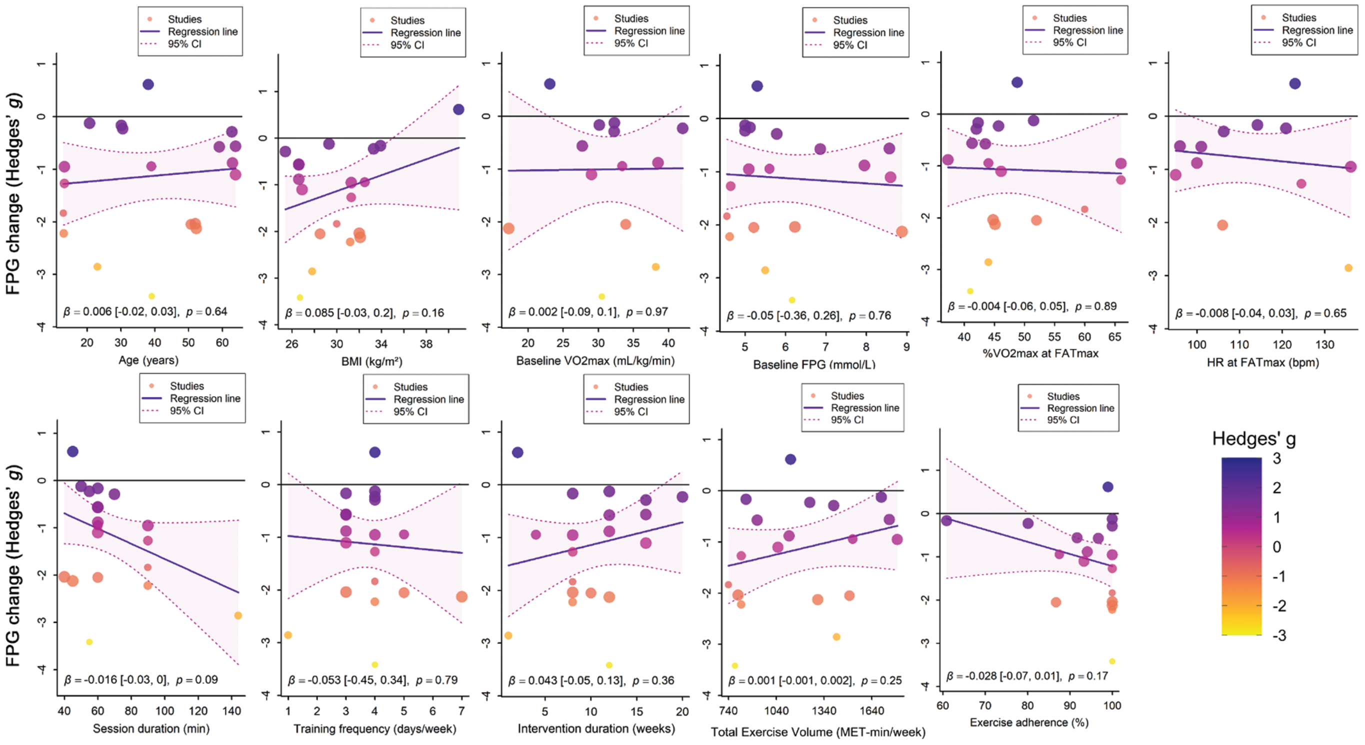

SUPPLEMENTARY FIG. 10. The bubble plots illustrate the relationship between FATmax-induced FPG changes (Hedges' *g*) and participant characteristics (Age, BMI, Baseline $\dot{V}O_{2max}$, Baseline FPG) or training parameters (%$\dot{V}O_{2max}$ at FATmax, HR at FATmax, Session duration, Training frequency, Intervention duration, Total Exercise Volume, and Exercise adherence). Each bubble represents an individual study, colored according to the magnitude of the effect size (see color scale on the right). The vertical axis represents the change in FPG. The solid purple line indicates the linear regression slope, while the pink shaded area (bounded by dotted lines) represents the 95% confidence interval (CI). The regression coefficient (β), its 95% CI, and the p-value are reported within each panel.

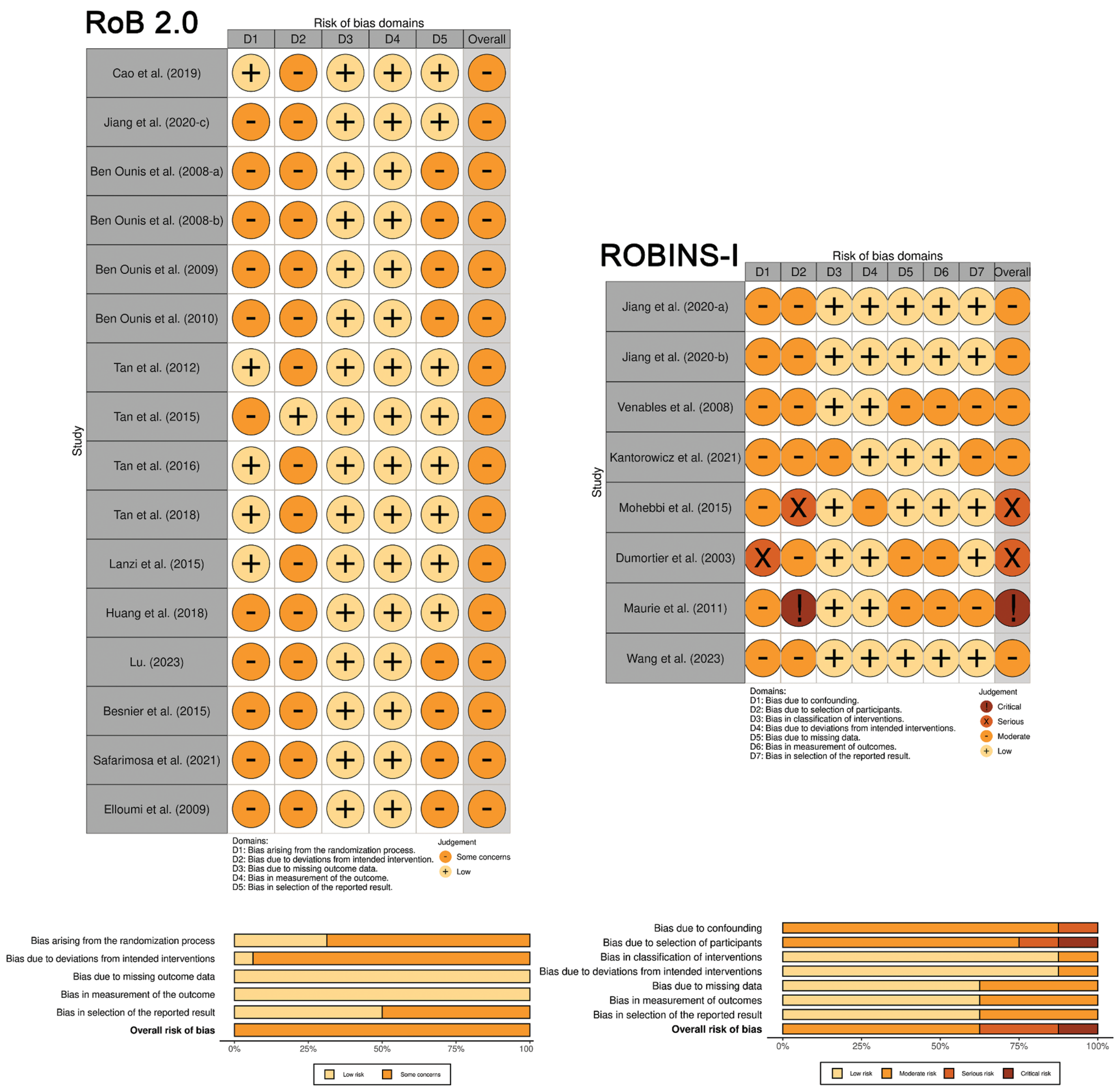

SUPPLEMENTARY FIG. 11. Risk of bias for the included studies

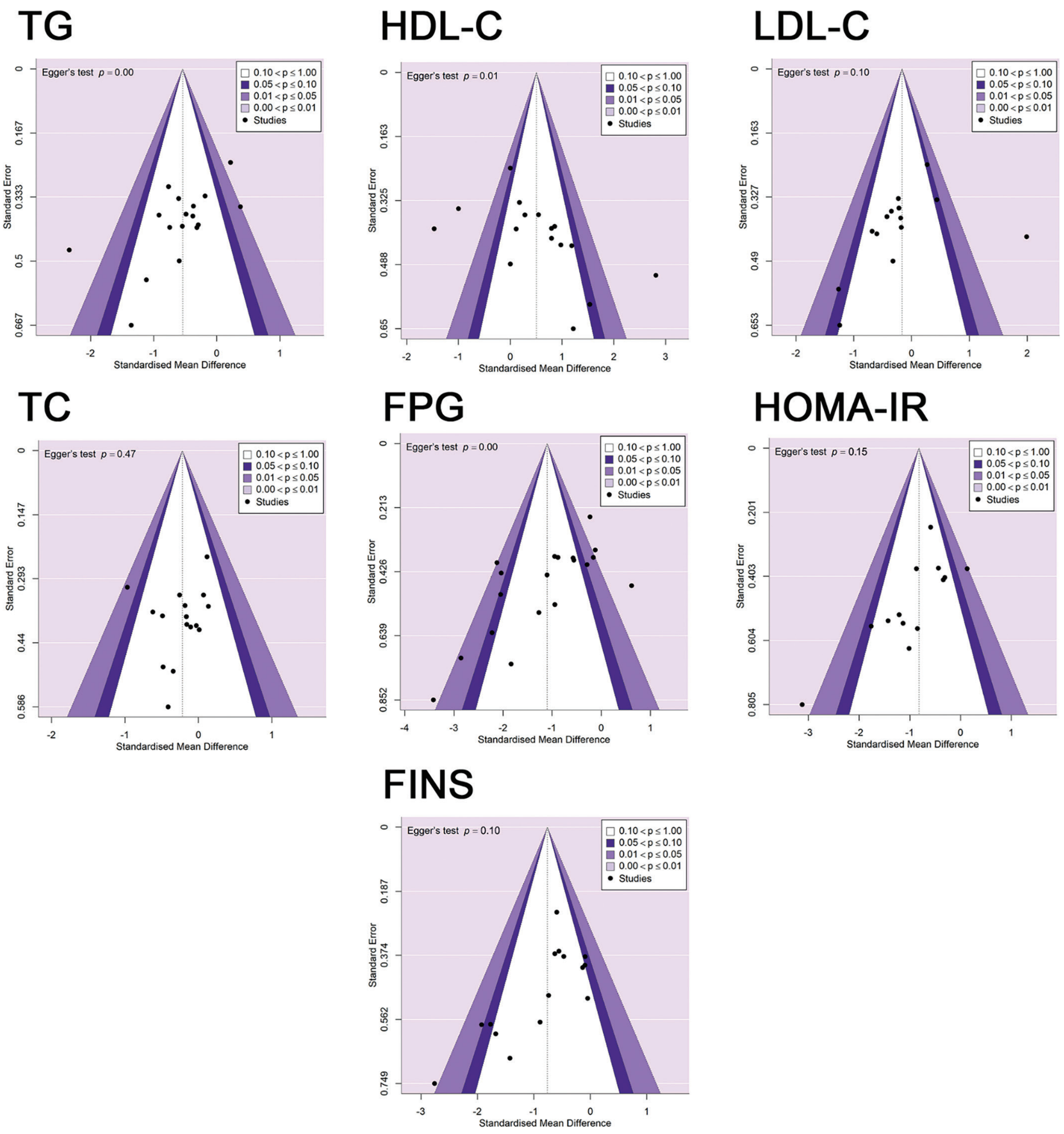

SUPPLEMENTARY FIG. 12. Funnel plot of included studies

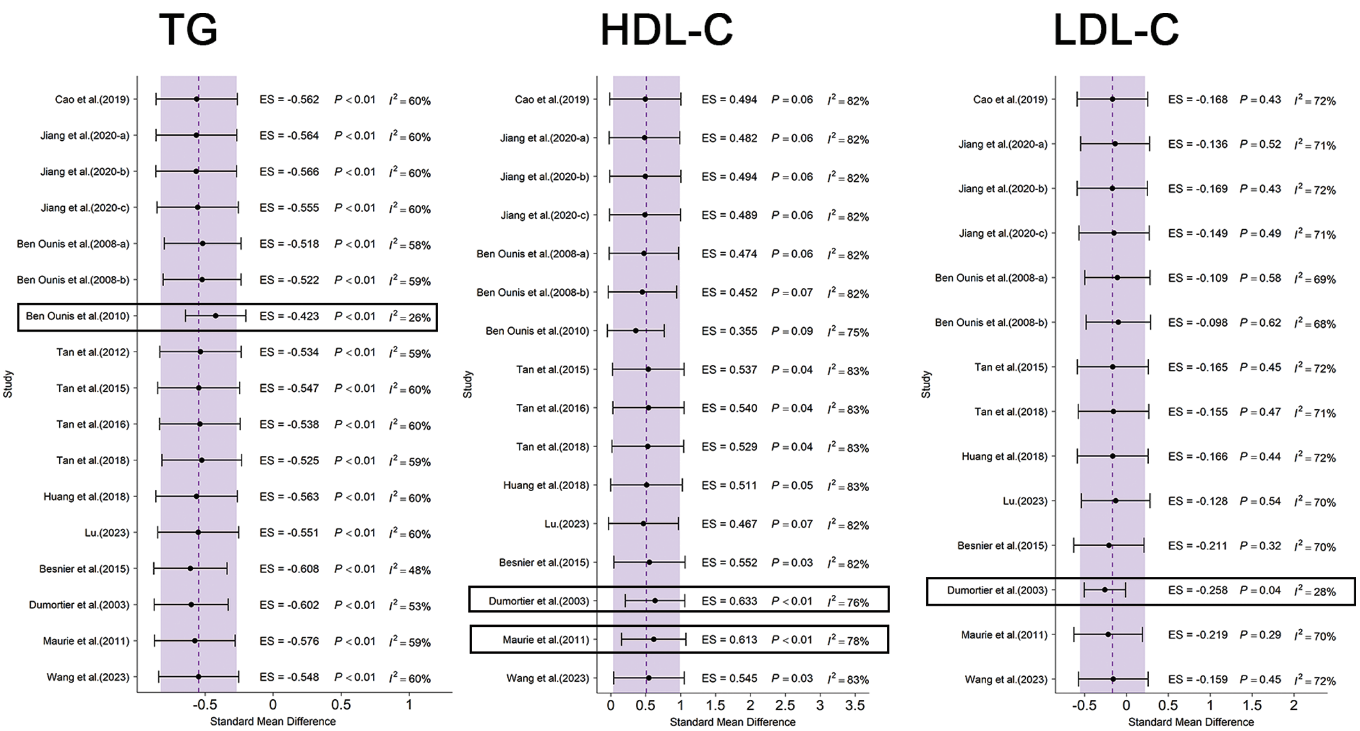

SUPPLEMENTARY FIG. 13. A sensitivity analysis based on leave-one-out

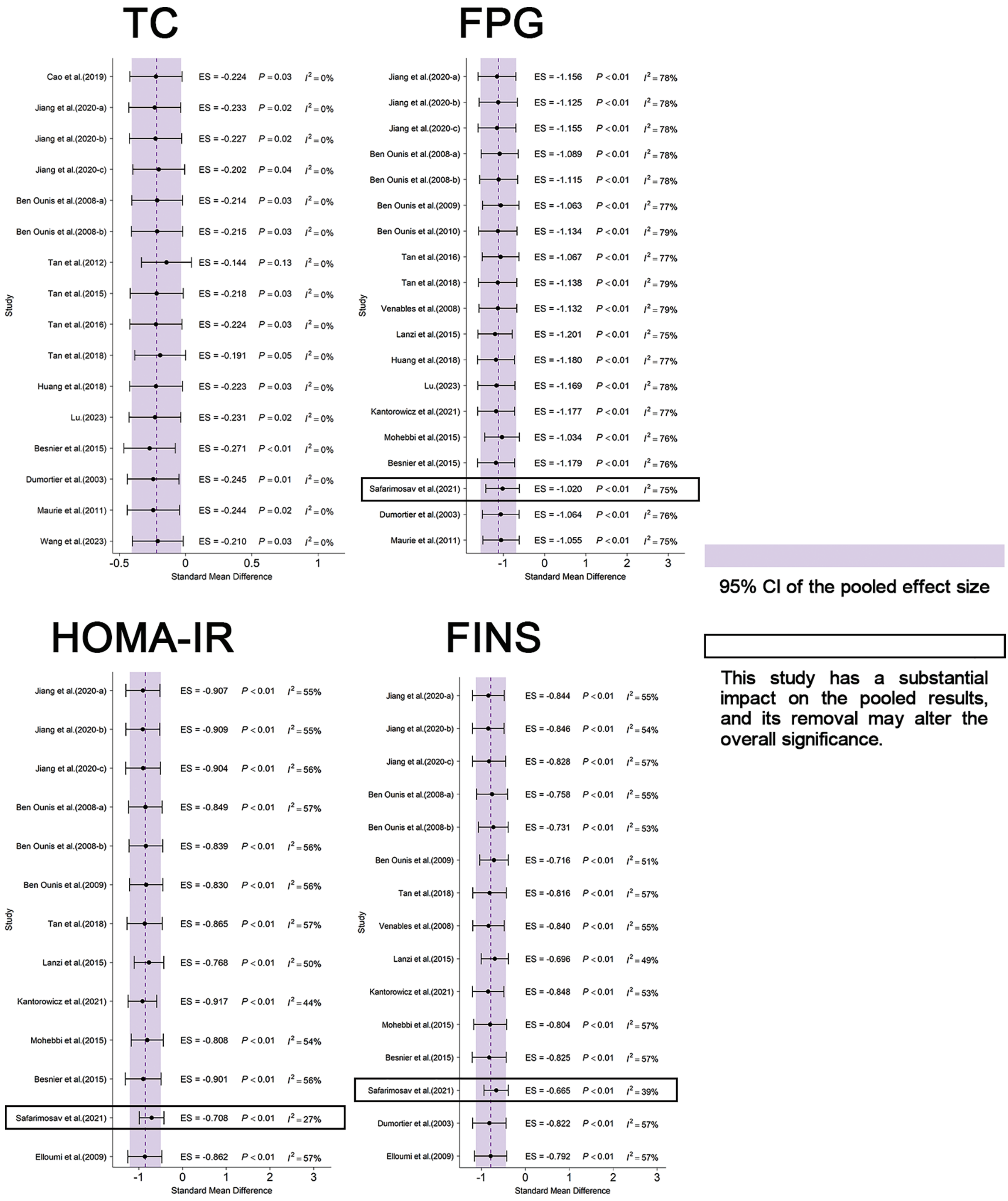

SUPPLEMENTARY FIG. 14. A sensitivity analysis based on leave-one-out

Articles published in the Biology of Sport are licensed under an open access Creative Commons CC BY 4.0 license.
